# Interface-Induced Concentration Enhancement in Glycine Solutions Investigated Using Surface Plasmon Resonance Spectroscopy and Molecular Dynamics Simulations

**DOI:** 10.1021/acs.jpclett.5c03688

**Published:** 2026-05-04

**Authors:** Ruairidh Mackay, Mozhdeh Mohammadpour, Binoy Paulose Nadappuram, Karen Johnston, Jan Sefcik, King Hang Aaron Lau

**Affiliations:** † EPSRC Future Manufacturing Research Hub in Continuous Manufacturing and Advanced Crystallisation, Technology and Innovation Centre, 3527 University of Strathclyde, Glasgow G1 1RD, U.K.; ‡ Department of Chemical and Process Engineering, 3527 University of Strathclyde, Glasgow G1 1XJ, U.K.; § Department of Pure and Applied Chemistry, 3527 University of Strathclyde, Glasgow G1 1XL, U.K.

## Abstract

Recent molecular simulations indicate nanoscale heterogeneity at liquid–solution interfaces, which is expected to significantly impact interfacial processes, such as catalysis and nucleation. We investigate the enhancement of glycine concentration in aqueous solutions at solid interfaces using molecular dynamics (MD) simulations and surface plasmon spectroscopy (SPR) measurements. MD predicts an interfacial concentration enhancement in a nanoscale solution region at the solid surface. SPR shows for the first time direct experimental evidence of such an enhancement in small molecule liquid mixtures. The excess interfacial mass density on gold and polystyrene surfaces for undersaturated glycine solutions reaches up to ∼50 ng/cm^2^, corresponding to a 1 nm layer with over double the glycine saturation concentration. We attribute the interfacial enhancement to omnipresent van der Waals interactions between solution components and surfaces. We expect this effect to be a common phenomenon in solutions that is likely to have profound effects on both natural and industrial interfacial processes.

## Graphical abstract

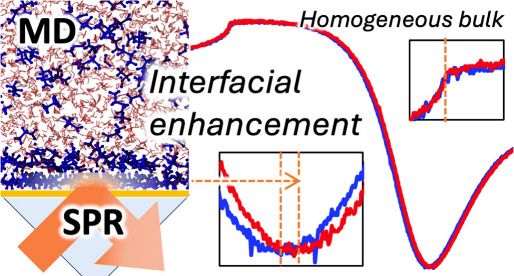

Many phenomena involving liquid solutions are driven by processes at the solution interfaces. For example, primary nucleation of crystalline solids from solutions typically proceeds via heterogeneous mechanisms, where the presence of an interface facilitates the birth of crystal nuclei—the surface energy of crystallites in contact with the interface is reduced and this decreases the size of the smallest stable (or critical) nucleus.^1^ Experimental studies of crystal nucleation have extensively explored interfacial effects based either on surface structure or topography^2,3^ or on surface functionalization^4,5^ rather than the intrinsic properties of a material in contact with a solution.^4,6^

It was recently reported that glycine nucleation is rapidly accelerated at solid (PTFE) and liquid (tridecane) hydrophobic surfaces in contact with aqueous glycine solutions.^6–8^ This was unexpected because these surfaces do not have a specific affinity with glycine, and the observations were mainly attributed to van der Waals interactions, which in turn led to the formation of a nanoscale layer of highly concentrated glycine solution at hydrophobic interfaces.^7^ Interfacial concentration heterogeneity has further been observed in MD simulations of liquid mixtures at solid surfaces, such as epoxy–amine mixtures at iron oxide surfaces,^9^ and binary Cu–Ni alloy liquid–solid interfaces,^10^ and is consistent with theoretical predictions of an interfacial concentration in binary metallic systems.^11,12^ Experimental studies of binary metal alloy interfaces^13^ and polymer solutions at solid surfaces^14^ have been reported to show interfacial heterogeneity and surface enrichment. Enrichment of some small molecule mixtures at liquid–vapor interfaces has been observed from direct measurements, such as amino acids at water–air interfaces^15^ and water–alcohol mixtures at air interfaces.^16,17^ However, to the best of our knowledge, an interfacial concentration enhancement effect for small molecules in liquid solutions at solid surfaces has not been directly observed experimentally or quantified previously. Moreover, such an interfacial effect challenges the widely held assumption of a uniform solution composition throughout the solution, for example within classical nucleation theory.^1^

In this work, we performed surface plasmon resonance (SPR) spectroscopy measurements and MD simulations of glycine solutions in contact with a solid surface to characterize and verify the interfacial enhancement effect. SPR is an in situ experimental surface technique conventionally used to measure the formation of molecular layers and the binding of various bio­(macro)­molecules within a nanoscale liquid region adjacent to a surface.^12,18–24^ We hypothesize that SPR should be extremely sensitive to interfacial concentration changes because, on a fundamental level, the technique relies on generating a surface-bound evanescent optical field on the SPR sensor chip (i.e., a surface plasmon) to detect refractive index (*n*_r_) changes around the surface, and *n*_r_ is strongly modified by the concentration of molecules dissolved in a solution.^18,25^

The paper is organized as follows: first we present MD simulations of a 250 g/kg solution (i.e., 250 g glycine in 1 kg water) in contact with a solid surface. We then present SPR measurements for glycine solutions with bulk concentrations ranging from 50 to 250 g/kg in contact with the native gold (Au) surface of SPR sensors as well as sensors coated with polystyrene (PS). The highest concentration was chosen to be slightly supersaturated at the experimental temperature (22 °C). All other experimental concentrations would remain undersaturated to investigate thermodynamically stable solutions and to highlight the magnitude of the interfacial effect. The SPR measurements verify the MD predictions and provide the first direct experimental evidence of the interface-induced concentration enhancement effect in small-molecule liquid mixtures.

MD simulations investigated the distribution of glycine and water molecules within a film of this solution in contact with a solid surface on one side and with a vacuum on the other side, as shown in the snapshot in Figure 1. The films ranged in thickness from 2.55 to 12.95 nm depending on the number of molecules in the system, while maintaining the same ratio of glycine to water corresponding to 250 g/kg glycine (see Computational Methods, Table 1). The surface was a generic Lennard-Jones (LJ) wall potential to illustrate the generality and magnitude of the interfacial effect. For clarity, “film” refers to the whole solution from solid surface to vacuum, and any interfacial region of enhanced glycine concentration found is described as a “layer”.

**Figure 1:**
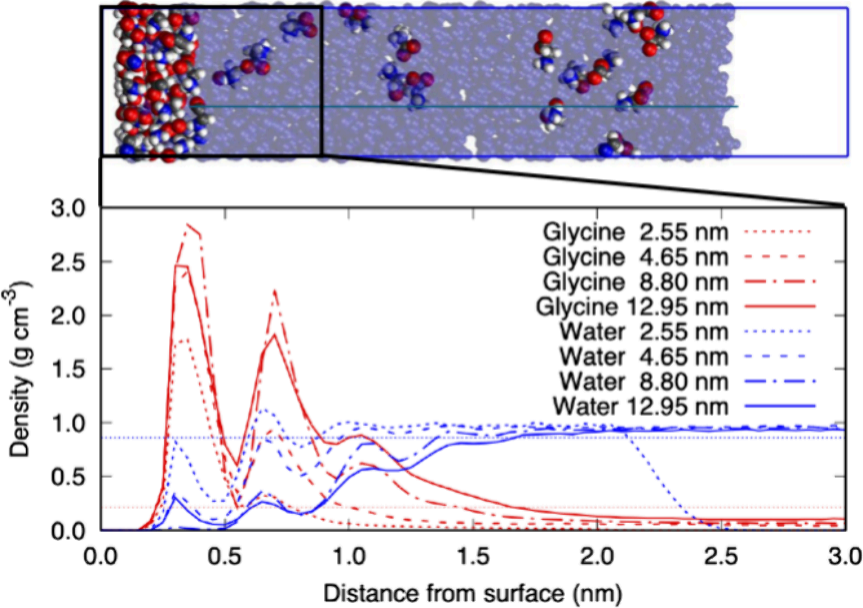
Snapshot of the simulation of the 8.80 nm film (top) and the density profiles of glycine (red) and water (blue) obtained from all of the simulations (bottom). The black-lined box bounding the left side of the snapshot highlights the region between 0 to 3 nm from the solid surface. The averaged density vs distance profiles of glycine and water in this region are plotted below for all film thicknesses (2.55 to 12.95 nm) simulated. The dotted blue and red horizontal lines indicate the water and glycine densities in bulk solution.

**Table 1:** System Configurations for the MD Simulations

Glycine Molecules	Water Molecules	Total Molecules	Simulation box length (nm)	Solution film width (nm)
15	250	265	4.05	2.55
30	500	530	6.10	4.65
60	1000	1060	10.20	8.80
90	1500	1590	14.30	12.95

The buildup of such a layer of glycine molecules at the surface can be clearly seen in the snapshot of Figure 1, which corresponds to the simulation of a 8.80 nm film of aqueous glycine, where the wall–solution interface is located on the left and the vacuum–solution interface is on the right). This effect is similar to previous simulations of higher concentration (supersaturated) glycine aqueous solutions at a vacuum-, liquid-, or solid-solution interface.^6,7^ The figure also shows the averaged glycine and water density profiles with the distance from the surface after equilibration. Two strong glycine peaks less than 1.0 nm from the surface are seen, and a weaker peak appears between 1.0 and 1.5 nm. The density profile of water shows a clear depletion of water molecules, confirming the glycine concentration enhancement.

We note that the interfacial concentration effect observed here could be different for other solution-surface combinations, as it will depend on the relative van der Waals interactions of the solute and solvent molecules with each other as well as with the surface, in conjunction with interfacial entropic effects. For example, for glycine aqueous solution at a vacuum (air) interface there is a depletion of glycine at the interface,^6^ whereas for ethanol–water mixtures there is an enrichment of ethanol molecules at the vacuum (air) interface.^26^ For this reason, we cannot a priori say whether a given solute–solvent surface will exhibit an enhancement or depletion at the surface. Nevertheless, our current understanding is that in glycine aqueous solutions, it is glycine’s stronger van der Waal’s interaction, compared to water, that is the primary driving force for the interfacial enhancement of glycine at the surfaces studied.

As the number of glycine molecules available is higher in thicker films, we see that the excess glycine extends further away from the surface and the thickness of the interfacial glycine concentration enhancement layer is increased. Far from the surface, the solutions are depleted in glycine. It is clear that even for the thickest films studied, the amount of glycine at the interface has still not reached the maximum value, which would require a sufficiently large reservoir of molecules representing the bulk solution. For the thickest films studied here (12.95 nm), the glycine interfacial concentration averaged over a 1 nm region is 1.0 g cm^–3^, as compared to the glycine crystal density of approximately 1.6 g cm^–3^.^27^ This indicates that for a strong wall potential, the interfacial concentration enhancement effect is substantial. However, the effect of a finite number of simulated glycine molecules makes the quantification of an interfacial layer thickness difficult. Nevertheless, the magnitude of increase in glycine interfacial concentration indicated by MD would be expected to cause a sufficiently large difference in molecular density between the interfacial region and the bulk liquid and hence refractive index contrast for the concentration enhancement to be detected in SPR measurements.

Figure 2 panels a and b show representative SPR reflectivity angle scan spectra for ethanol (an “index-matched solvent”, see below) and for a glycine solution with bulk concentration of 176 g/kg, respectively, both measured on the native Au surface of an SPR sensor chip. Data analysis of the SPR spectra is aided by two key qualitative features. First, the total internal reflection angle (θ_TIR_), revealed by a reflectivity “edge” (insets for 49°-50.2°), solely depends on the bulk refractive index (*n*_r_) of the solution (*n*_r‑bulk_). In contrast, the plasmon coupling angle (θ_SPR_), indicated by the reflectivity minimum (insets for 62°-63.2°), depends additionally on the refractive index of any interfacial layer present (*n*_r‑interf_) and its thickness (*t*).^18^ As a departure from conventional SPR characterizing the surface binding of biomacromolecules from dilute solutions,^18,21–23,25^ for which the increase in *n*_r‑bulk_ due to dissolved solute molecules can be ignored, *n*_r‑bulk_ of the 176 g/kg glycine solution is significantly higher than water (Table S1). We therefore paired the glycine solution measurement with a measurement of ethanol, which exhibits a refractive index closely matching 176 g/kg glycine (Tables S2 and S3 and Figure S1). Thus, the two spectra should have identical θ_TIR_ values, but the presence of an interfacial concentration enhancement layer and its influence on *n*_r‑interf_ would be revealed by a difference in θ_SPR_.

**Figure 2:**
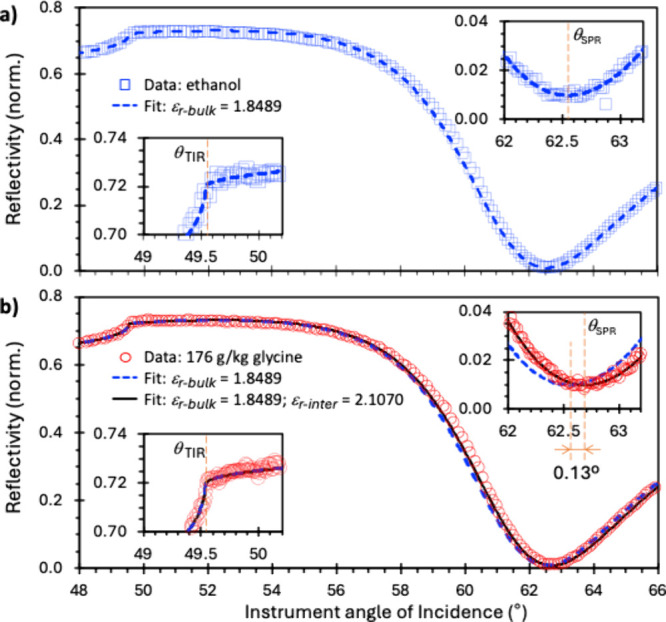
Representative SPR angle scan reflectivity spectra at a Au surface for (a) ethanol and (b) glycine solution with a 176 g/kg bulk concentration. Data are shown in symbols. Solid and dashed lines correspond to calculated reflectivity fits with and without an assumed 1 nm thick interfacial layer, respectively. The insets for 49–50.2° show that the total internal reflection angle (θ_TIR_) is the same for both samples due to refractive index matching. The insets for 62–63.2° show that the glycine solution has a higher SPR coupling angle (θ_SPR_), indicating the presence of an interfacial glycine layer with a higher local concentration and *ε*_r‑interf_ > *ε*_r‑bulk_.

Accordingly, Figure 2 shows that θ_TIR_ of the two liquids is essentially identical at an instrument angle of 49.53°, demonstrating the intended index matching to high precision. In contrast, θ_SPR_ = 62.68° for the glycine solution compared to 62.55° for ethanol (i.e., a 0.13° change, more than an order of magnitude larger than the 0.01° measurement steps and the 0.002° repeatability of our setup). This would be expected if, at the SPR sensor surface, there is a concentration of molecules with refractive index higher than the solvent, such as glycine in water. In contrast, the physical adsorption of molecules as small as glycine would generally result in layers too thin to be routinely detected by SPR.^18,28^ In other words, a comparison of the glycine solution and its index-matched spectra shows, for the first time, that there is a significant interfacial layer at the solid-solution interface that corresponds to an enhanced glycine concentration compared to the bulk solution.

To quantify the interfacial concentration enhancement phenomenon, we analyzed the ethanol and 176 g/kg glycine angle scans with the established “Winspall” optical model^18,20^ that relates θ_TIR_ and θ_SPR_ to *n*_r‑bulk_ and *n*_r‑interf_ (see overlaid lines in Figure 2 for calculated fits and SI Sections 6.5 and 6.6 for fitting procedure). In this model, the thickness of the interfacial layer was assumed to be 1 nm, approximating the order of magnitude of the effect indicated by MD. To facilitate later thickness independent analysis in terms of excess interfacial mass densities (see below), we describe all optical properties hereafter in terms of relative permittivity, *ε*_r_ = *n*_*r*_^2^.

The index-matched measurement in ethanol (Figure 2a) gives *ε*_r‑bulk_ = 1.8489, matching the literature value of 1.8507 to within 1% (Table S3). The glycine solution measurement (Figure 2b) indicates *ε*_r‑interf_ = 2.1070, which is consistent with a significantly higher glycine concentration at the interface than in the bulk (*ε*_r‑bulk_ = 1.8489, identical with that of the index-matched ethanol).

To verify the interfacial enhancement and further characterize the generality of the phenomenon, we repeated measurements at 176 g/kg glycine and also expanded the bulk concentration studied to 50 to 250 g/kg glycine using the same as well as different SPR Au chips (N = 9–13 repeats across 11 chips, Tables S1–S4). Concentrations below the saturation limit were chosen to rule out any crystallization of glycine on the surface. To provide refractive index-matching across the glycine concentrations, measurements of a range of methanol-propanol mixtures were also performed (see SI Section 6.3, Table S2 and Figure S1). We further repeated the measurements on sensor chips spin coated with a polystyrene (PS) organic layer, which represents a material chemically distinct from Au, across all glycine concentrations (*N* = 10–12, Tables S2, S4 and S5).

The averaged *ε*_r‑interf_ and *ε*_r‑bulk_ results (i.e., *ε̅*_r‑interf_ and *ε̅*_r‑bulk_) on Au and PS surfaces are plotted in Figure 3 panels a and b, respectively, as functions of the bulk solution concentration (see Tables S6 and S7 for values). On Au, it is seen that *ε̅*_r‑interf_ increases gradually with increasing glycine concentration, reaching *ε̅*_r‑interf_ = 2.0717 ± 0.0334 at 176 g/kg glycine, bracketing the data shown in Figure 2. The values at higher glycine concentrations are similar. In contrast, *ε̅*_r‑bulk_ steadily increases with the bulk solution concentration, as expected, but *ε̅*_r‑interf_ remains consistently higher than *ε̅*_r‑bulk_ at any given concentration, indicating the presence of an interfacial concentration enhancement layer in all cases.

**Figure 3:**
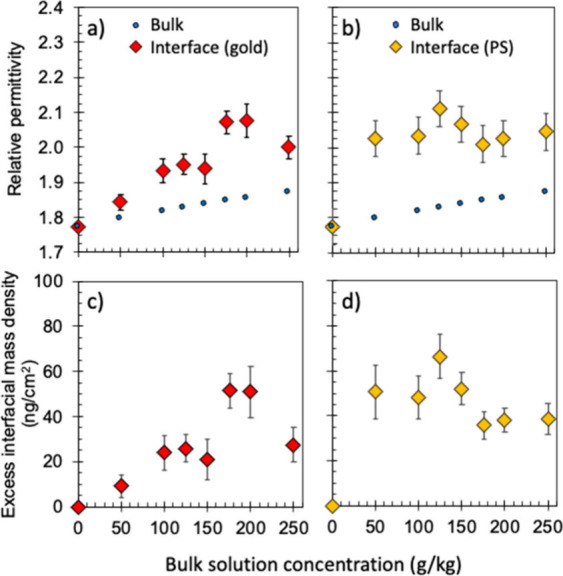
Averaged relative permittivity within the interfacial solution layer (*ε̅*_r‑interf_) plotted as a function of bulk solution concentration at a) the Au (red diamonds) and b) the PS (yellow diamonds) surfaces. The results are based on an interfacial layer thickness of 1 nm and are compared with the averaged measured bulk solution relative permittivity (*ε̅*_r‑bulk_, blue circles). The error bars represent the standard error of the mean (error values for *ε̅*_r‑bulk_ are smaller than the data symbols and are obscured). The excess interfacial mass density (Γ) calculated from *ε̅*_r‑interf_ is shown in c) for Au and d) for PS (see SI Sections S4 and S5 for details).

On PS, the same *ε̅*_r‑bulk_ values as on Au were measured, and *ε̅*_r‑interf_ is also consistently higher than *ε̅*_r‑bulk_. This verifies both the generality of the interfacial layer and its independence from bulk properties. Unlike on Au, a high *ε̅*_r‑interf_ around 2 was already observed on PS from the lowest 50 g/kg to the highest 250 g/kg bulk glycine concentration investigated. The different trends between Au and PS likely reflect different strengths of van der Waals forces on these materials. The lack of a further increase in *ε̅*_r‑interf_ also suggests that there may be a maximum extent to the interfacial enhancement, depending on the properties of the solute molecule (i.e., glycine).

The variance observed in the data, represented in Figure 3a and b by error bars showing ±1 standard error of the mean (SEM), is expected for measurements of such thin solution layers on the order of 1 nm. While we saw chip-to-chip variation in the *ε̅*_r‑interf_ values measured (see Figures S2 and S3), possibly due to intrinsic variations of individual SPR chips (e.g., differences in Au and PS *ε*_r_ and *t* values, Tables S4 and S5), the variance across repeated measurements on a given chip were always smaller than the difference between *ε̅*_r‑bulk_ and *ε̅*_r‑interf_ for that surface at any particular concentration (Tables S6 and S7). Moreover, a high data quality was demonstrated by the essentially identical values of *ε̅*_r‑bulk_ measured between measurements of different liquid compositions across different chip samples and surface materials (differences ≲ 0.1%) (Tables S1 and S2) as well as by the good agreement of *ε̅*_r‑bulk_ with values reported in the literature (Figure S1). Furthermore, there was no systematic difference observed when the measurement order of the various concentrations was switched. These observations indicated the reversible nature of the interfacial enhancement layer and that the effects of residue or accumulated adsorption, if any, were small.

To more easily discern the interfacial concentration enhancement against the high concentration background of the glycine bulk solution and to quantify the enhancement without assuming a thickness value of the layer, we related the SPR results to the excess interfacial mass density of solute molecules (Γ), i.e., the interfacial glycine mass density per unit surface area in excess of the bulk solution. Γ was estimated by first relating *ε̅*_r‑interf_ to the mass fraction of glycine in the solution (*x*_gly_) through the Clausius-Mossotti equation (see SI Section S4 and Figure S4a). Then, interfacial mass densities were calculated from the volumetric mass densities corresponding to *x*_gly_ according to interpolation of literature density data measured for glycine solutions and pure glycine and water (Figure S4b) (see SI Section S5 for example calculation). The excess interfacial mass density is invariant with respect to the thickness assumed in the SPR Winspall model—a larger assumed thickness would be compensated by a lower *ε*_r‑interf_ to account for the same θ_SPR_ shift measured and vice versa.

Figure 3 panels c and d show the trends in Γ at the Au and PS surfaces, respectively, that parallel the trends in *ε̅*_r‑interf_. This is expected since both Γ and *ε*_r‑interf_ are related through *x*_gly_. On Au, Γ gradually increases to ∼50 ng/cm^2^ as the bulk glycine concentration increases to 150 g/kg and beyond. A similar maximal value of Γ is obtained on PS but this is already achieved from 50 g/kg bulk glycine, and it remains roughly unchanged through to the higher bulk concentrations studied. An interfacial excess of Γ = 50 ng/cm^2^ is equivalent to 5 × 10^–13^ ng/nm^2^ which, if concentrated within a 1 nm interfacial layer would imply ∼4 additional glycine molecules confined within 1 nm^3^. This excess glycine concentration would be significantly over and above the 1.7 glycine molecules per 1 nm^3^ of a corresponding bulk solution at 250 kg/kg, which should already be slightly supersaturated at the experimental temperature of 22 °C.^33^ In comparison, crystalline glycine has a density of ∼1.6 g/cm^3^ (see SI Table S8), equivalent to 16 ng/nm^3^ or ∼13 glycine molecules per 1 nm^3^. Thus, an interfacial enhancement layer thickness of 1 nm implies a relatively dense interfacial region that is roughly one-third of the molecular density of a crystalline phase, depending on the molecular packing, and over twice the molecule density in a saturated solution.

In conclusion, we investigated glycine concentration enhancement in undersaturated aqueous solutions at solid–liquid interfaces using MD simulations and in situ surface measurements using SPR. Simulations predicted the formation of a nanoscale region of a dense glycine-rich solution layer in the vicinity of the surface. SPR measurements provided the first direct experimental evidence of significant interfacial concentration enhancement in undersaturated glycine solutions at all bulk solution concentrations between 50 and 250 g/kg at both the Au and PS interfaces. The excess interfacial mass densities increased with increasing solution concentration, reaching a maximum of ∼50 ng/cm^2^ on Au surfaces seen from 150 to 250 g/kg glycine in the bulk. Similar excess interfacial mass densities were obtained on PS interfaces across the range of glycine concentrations investigated.

While SPR is unable to decouple the concentration of the interfacial layer and its thickness, insight from MD simulations enables us to deduce that the interfacial region is on the order of a nanometer, although we note that the MD simulations show a depletion of glycine due to the finite size of the simulations, which limits the concentration enhancement. Future work should focus on quantifying this phenomenon for a wide range of systems of scientific and industrial significance. An interface-induced concentration enhancement, as observed here, is expected to be a generic phenomenon in liquid solutions, which has clear implications for interfacial processes such as fouling prevention, biosensing, and heterogeneous nucleation and catalysis.

## Experimental Methods

Details of materials and sample preparation methods are described in SI Section 6. All glass and other labware used were cleaned by sonication in a Hellmanex solution and extensive rinsing. SPR Au sensor chips were prepared by vapor deposition on double-sided quarter wavelength flat glass slides. A purpose built SPR setup was used to acquire reflectivity angle scans. The temperature during measurements was 22 ± 0.23 °C (Table S1). Glycine solutions were prepared at concentrations of 50, 100, 125, 150, 176, 200, and 250 g/kg glycine in DI water in glass vials (concentrations prepared within 0.05% of target, see Table S1). Binary mixtures of methanol and 1-propanol were prepared as index matched liquids for all glycine solutions except for the 176 g/kg solution, which was matched using pure ethanol (Table S1). Samples were checked for surface defects by brightfield and darkfield microscopy. Multiple repeated measurements were performed for each solution condition across different SPR chips (*N* = 9–13 across at least 10 Au chips and 6 PS coated chips), and the averages and standard errors are reported (see Tables S1–S7). Angle scans followed a defined sequence, whereby measurements of index-matched samples were undertaken prior to and in between measurements of glycine solutions. For each measurement, the liquid cell was flushed with DI water three times and further flushed three times with the sample of interest before refilled a fourth time and held for 3 min before the measurement. SPR spectra were analyzed using Winspall,^29^ a freely available software widely used for >20 years,^18–20,24^ to determine *ε*_r_ and thickness of each component in the system (see SI Section 6 for procedures).

## Computational Methods

MD simulations were performed using LAMMPS software (22 July 2025).^30^ Glycine was represented using the generalized AMBER Force Field (GAFF) with CNDO charges, and water was represented using the SPC/E water model as in previous studies.^7,31^ The model surface was represented by a Lennard-Jones (LJ) 9–3 wall potential with σ_LJ_ = 0.34 nm and *ε*_LJ_ = 10.0 kcal mol^–1^, which are within the range of LJ parameters determined for heptane, tridecane, and graphite surfaces.^6^ The van der Waals interaction parameters for the solvent and solute with the LJ 9–3 wall were determined using modified Lorenz-Berthelot mixing rules, as described in our previous work.^6^ All LJ interactions, including the LJ 9–3 wall potentials, were truncated at a cut off of 1.4 nm. Short-range electrostatic interactions were calculated up to a distance of 0.98 nm, whereas long-range electrostatic interactions were evaluated using a particle–particle particle-mesh method. The LAMMPS input files are provided with open access (see Data Availability).

For simulations of glycine films in contact with the model surface, glycine, and water molecules were randomly inserted into a tetragonal box, with a cross-sectional area 2.05 nm × 2.05 nm in the *x*–*y* plane. A vacuum region of approximately 1.5 nm was left at one end of the box. Different film thicknesses and numbers of molecules were simulated, while preserving the concentration at 250 g/kg, as summarized in Table 1, shown below. Periodic boundary conditions were applied in the *x* and *y* directions, while nonperiodic boundaries were implemented in the *z*-direction with the model surface on one side of the glycine film and the vacuum layer on the other side. A slab correction was used for the PPPM solver to account for nonperiodicity of the system in the *z*-direction. The system was energy minimized and then simulated using an NVT ensemble. The NVT simulations used a Nosé–Hoover thermostat with a damping parameter of 100 fs to maintain a temperature of 298 K. A time step of 2.0 fs was used in all of the simulations. The small to large films were run for 40, 60, 100, and 190 ns, respectively.

The density profiles were analyzed as a function of the distance from the LJ wall in 0.05 nm bins. The interfacial concentration profiles were analyzed over the final 20 ns, 40 ns, 60 and 50 ns for the 15, 30, 60, and 90 glycine molecule systems, respectively. The glycine solutions occupied the part of the simulation box adjacent to the surface and had widths shown in Table 1, which were estimated based on when the density of water fell to less than 0.1 g cm^–3^ for the analyzed trajectories. Simulations were visualized using VMD.^32^

## Supplementary Material





## Data Availability

Sample input files for the LAMMPS molecular dynamics simulations described in this publication are openly available from the University of Strathclyde Knowledge Base at 10.15129/e31ae64a-1a03-4c84-b9ba-274ab2cc7929.

## Notes

SPR measured permittivity values, literature reference values used, details on estimating the correspondence between permittivity and mass fraction, and materials and experimental methods (PDF)
Transparent Peer Review report available (PDF)
