# Supplementary material for: Interface-Induced Concentration Enhancement in Glycine Solutions Investigated Using Surface Plasmon Resonance Spectroscopy and Molecular Dynamics Simulations
Source: J Phys Chem Lett. 2026 May 4;17(19):5535–40. doi: 10.1021/acs.jpclett.5c03688 (PMC13181769; doi:10.1021/acs.jpclett.5c03688)
Supplement: Supplementary file 1 [file jz5c03688_si_001.pdf]

Supporting Information:

Interface-Induced Concentration Enhancement in Glycine Solutions Investigated Using Surface Plasmon Resonance Spectroscopy and Molecular Dynamics Simulations

Ruairidh Mackay,[1,2,3] Mozhdeh Mohammadpour,[2] Binoy Paulose Nadappuram,[3] Karen Johnston,[2] Jan Sefcik,[1,2]* King Hang Aaron Lau[3]**

[1] EPSRC Future Manufacturing Research Hub in Continuous Manufacturing and Advanced Crystallisation, Technology and Innovation Centre, University of Strathclyde, Glasgow G1 1RD, UK.

[2] Department of Chemical and Process Engineering, University of Strathclyde, Glasgow G1 1XJ, UK.

[3] Department of Pure and Applied Chemistry, University of Strathclyde, Glasgow G1 1XL, UK.

*Corresponding Authors:
Karen Johnston karen.johnston@strath.ac.uk, Jan Sefcik jan.sefcik@strath.ac.uk, King Hang Aaron Lau aaron.lau@strath.ac.uk

Table of Contents

S1. SPR data for glycine solution and reference solvent measurements..5
S2. Measurement data of glycine solutions on Au and PS sensor chips..10
S3. Permittivities measured for individual sample chips 11
S4. Estimating εr of mixtures based on the Clausius-Mossotti equation 13
S5. Estimating excess interfacial mass density (Γ)..18
S6. Detailed Experimental Methods 20
S7. Molecular Simulations Detail...23
References..24

Table of Figures

Figure S1. *Comparison of measured bulk permittivities of both pure solvents and index-matched methanol:1-propanol (MeOH:1PrOH) mixtures with literature values, the values obtained from the Clausius-Mossotti equation [9] (see Section S4) and the values from an arithmetic interpolation of the pure MeOH and 1PrOH solvents. The temperature recorded during the measurements was between 21.4-22.3 ºC (mean = 21.8 ± 0.24) and the measurements were based on the critical total internal reflection angle of a 632.8 nm HeNe laser. See **Table S2** for the plotted values and standard deviations at each condition. See **Table S3** for literature reference values. The Clausius-Mossotti equation predicts $\varepsilon r - bulk$ values closer to the experimental values than a simple linear interpolation, although the values are still slightly too high due to the assumption of ideal molecular mixing. Nonetheless, the differences are <0.3%.* 8

Figure S2. *Plots of $\varepsilon r - interf$ and $\varepsilon r - bulk$ measured at different bulk glycine concentrations on each individual gold coated SPR sensor chips (Au chips). The temperature recorded during the measurements was between 21.5-22.3ºC. See **Table S1** for $\varepsilon r - bulk$ values and **Table S6** for mean values and the standard deviations and standard errors of $\varepsilon r - interf$. See **Table S4** for the thickness and permittivity values of Cr and Au for each SPR chip.* 11

Figure S3. *Plots of $\varepsilon r - interf$ and $\varepsilon r - bulk$ measured at different bulk glycine concentrations on each individual gold coated SPR sensor chips (Au+PS chips). The temperatures recorded during the measurements was between 21.4-22.3ºC. See **Table S1** for $\varepsilon r - bulk$ values and **Table S7** for mean values and the standard deviations and standard errors of $\varepsilon r - interf$. See **Table S4** for the thickness and permittivity values of Cr and Au, and **Table S5** for the permittivity values of thiol and PS, for each SPR chip.* 12

Figure S4 *a) Glycine mass fraction (x_{gly}) is estimated from the measured bulk $\varepsilon_{r\text{-}bulk}$ and interfacial $\varepsilon_{r\text{-}interf}$ (see **Table S1, Table S6** and **Table S7**, respectively, for values) through the Clausius-Mossotti equation (dashed line; see Section S4 and Equation 1 for details). The plot also compares these values with literature relative permittivity data of glycine solutions (Soto 1999 [22]). A linear interpolation (solid line) between literature $\varepsilon_{r\text{-}bulk}$ values of water (at x_{gly} = 0) and different glycine crystal polymorphs (x_{gly} = 1) is also shown for comparison (see **Table S3** and **Table S9** for values and references). b) The volumetric mass density (ρ) is related to x_{gly} through a polynomial interpolation (dotted line; $\rho = 0.2288\, x_{gly}^2 + 0.3905\, x_{gly} + 0.9976$) of literature data available for glycine solutions (Soto 1999 [22]) and for glycine crystal polymorphs (**Table S8**). In comparison, linear interpolations based on either fitting the literature glycine solution values (dahed green line) or the x_{gly} = 0 and 1 end point values for the density of water (**Table S11**) and the average of glycine polymorphs (solid green line) fail to account for all the literature values.* 17

Figure S5*. Schematic illustrating the dimensions of the interfacial layer with enhanced glycine concentration with thickness t and a 1 cm^2 unit area.* 19

Figure S6*. Diagram showing key components of the SPR spectrometer setup.* 21

Figure S7*. Schematic of the constituent layers used in the optical model analysis of the experimental SPR system. All measurements/models include glass prism, chromium (Cr), gold (Au) and bulk liquid layers.*

Measurements on polystyrene (PS) coated Au chips also include thiol and PS layers. Glycine solution measurements can also include an interfacial layer, if present. Each layer is described by its refractive index and thickness, except for the macroscopic glass and bulk liquid layers, which are described only by n_r values because of their sizes are macroscopic compared to the thin nanoscale interfacial layers. ______________ 22

Table of Tables

Table S1. Glycine solution concentrations (conc.) targeted and prepared, and corresponding values of mean relative permittivity ($\varepsilon r - bulk$), measured across "n" repeated number of measurementss. 5
Table S2. Bulk solvent relative permittivities ($\varepsilon r - bulk$) of reference solvents (methanol—MeOH, ethanol, 1-propanol—1PrOH, and water) and the mean measured bulk relative permittivities ($\varepsilon r - bulk$) of MeOH:1PrOH mixtures with permittivity values as close as possible to the targeted glycine (gly.) solutions shown in **Table S1**. The mean was obtained from "n" repeat measurements. Also shown are the MeOH:1PrOH volume percentages used to achieve this "index matching", and the percentage differences (diff.%) of $\varepsilon r - bulk$ from literature and corresponding glycine solution values. 5
Table S3. Literature values of refractive index (n) and calculated $\varepsilon r - bulk$ = n^2 for water, methanol, ethanol, and 1-propanol. In black are reference values with measurement wavelength and temperatures (632.8 nm, 21.5-22ºC) corresponding to the present study (632.8 nm, 21.4ºC - 22.3ºC). For comparison, in gray are literature values with either wavelength or temperature not matching the present study. 6
Table S4. The thickness and relative permittivity (εr) values of Cr and Au layers. There were three batches of SPR chips corresponding to metal layers deposited at different times. As expected in vapor deposition, the values within each batch closely matched with each other but the batch averages can vary. Matching of SPR data with Cr and Au parameters were significantly aided by the semi-independent ways that different parameters modify the reflectivity data: i) the Cr and Au thickness values have dominant control over the slopes after and before the θ_{TIR} angle, respectively; ii) the Cr layer is comparatively so much thinner than Au that variations in $\varepsilon r - Cr$ have little influence and a standard value consistent with the literature and past samples was used; and iii) the real and imaginary parts of $\varepsilon r - Au$ control the θ_{SPR} angle position and the width of the reflectivity minimum centered around θ_{SPR}. The (complex) n_r of the average Cr and Au permittivities are also shown and are similar to literature values [8]. The standard deviation (SD) of $\varepsilon r - Au$ refer to the small variations (0.5-3%) between measurements using the same SPR chip (see **Table S1** and **Table S2**). 7
Table S5. SPR measurement data values for the "thiol" (dodecanethiol SAM) and PS (spin-coated polystyrene) layers used for PS-glycine solution interface measurements. See **Table S4** for details on batch numbering and the Cr and Au parameter values for each SPR chip. 9
Table S6. SPR measurement data values for the Au-glycine solution interface. The mean concentrations, relative permittivity of the bulk liquid ($\varepsilon r - bulk$) and of a 1 nm interfacial region ($\varepsilon r - interf$) were measured across "n" repeated number of measurements. SD denotes standard deviation; SEM denotes standard error of the mean, i.e., the expected bounds of the average given the number of measurements, calculated as SEM = SD/ $\sqrt{n}$. The ±1 SEM is shown as error bounds in the main text and accompanying Figure 5a. 10
Table S7. SPR measurement data values for the PS-glycine solution interface. The mean concentrations, relative permittivity of the bulk liquid ($\varepsilon r - bulk$) and of a 1 nm interfacial region ($\varepsilon r - interf$) were measured across "n" repeated number of measurements. SD denotes standard deviation; SEM denotes standard error of the mean, i.e., the expected bounds of the average given the number of measurements, calculated as SEM = SD/ $\sqrt{n}$. The ±1 SEM is shown as error bounds in the main text and accompanying Figure 5b. 10
Table S8. Literature values of mass density (ρ) for various glycine crystals. The average value was used for calculation of the volumetric number density, N_{gly} (see Section S0 for details). 14
Table S9. Literature values of refractive index (n) and the calculated bulk relative permittivity ($\varepsilon_{r\text{-}bulk}$) for various glycine crystals. The average value was used for calculation of α_{gly}. The permittivity is related to the refractive index by ε_{bulk} = n^2. 14

Table S10. *Literature values of the volumetric mass density (ρ) for methanol and 1-propanol. Data for a temperature matching the present measurement (22ºC, see Figure S1 caption) was not found. As an approximation, literature densities from 16.85ºC , 20ºC , 25ºC and 26.85ºC were interpolated, and these were averaged to obtain an estimate corresponding to 22ºC.* *15*
Table S11. *Values of molecular polarizability (α) at 22ºC based on the Clausius-Mossotti equation and corresponding literature values of the volumetric mass density (ρ), number density (N), and relative permittivity (ε_r). See Section S4, ref.10,* ***Table S8*** *and* ***Table S10*** *for details.* *15*
Table S12. *Correspondence between bulk glycine solution concentration (glycine mass/1 kg water) and the glycine mass fraction (x_{gly} = glycine mass/(glycine mass + water mass)) for the concentration values used in this study. The corresponding volumetric mass density, estimated from fitting (**Figure S4**) of literature values [22], is also shown.* *16*

S1. SPR data for glycine solution and reference solvent measurements

Table S1. *Glycine solution concentrations (conc.) targeted and prepared, and corresponding values of mean relative permittivity ($\bar{\varepsilon}_{r-bulk}$), measured across "n" repeated number of measurementss.*

n (Au)	n (Au +PS)	Target conc. (g/kg)	Actual conc. (mean of n repeats) (g/kg)	SD[a] of conc. (g/kg)	SD of conc. (%)	Difference of conc. from target (%)		$\bar{\varepsilon}_{\text{r-bulk}}$ [b,c] (unitless)	SD of $\bar{\varepsilon}_{\text{r-bulk}}$	SD of $\bar{\varepsilon}_{\text{r-bulk}}$ (%)
10	12	0	Na	na	na	na		1.7732	0.0001	0.00%
9	11	50	50.00	0.0193	0.04%	-0.01%		1.7966	0.0010	0.06%
9	12	100	99.97	0.0372	0.04%	-0.03%		1.8175	0.0006	0.03%
9	11	125	125.06	0.0514	0.04%	0.05%		1.8270	0.0010	0.05%
9	12	150	149.97	0.0522	0.03%	-0.02%		1.8370	0.0008	0.05%
18	12	176	175.94	0.0560	0.03%	-0.03%		1.8468	0.0006	0.03%
13	10	200	200.00	0.0927	0.05%	0.00%		1.8555	0.0008	0.04%
9	11	250	249.99	0.0995	0.04%	0.00%		1.8722	0.0017	0.09%

[a] SD denotes standard deviation; [b] A stroke on top of the symbol for relative permittivity (ε_r) denotes the mean; [c] The refractive index is square root of ε_r.

Table S2. *Bulk solvent relative permittivities (ε_{r-bulk}) of reference solvents (methanol—MeOH, ethanol, 1-propanol—1PrOH, and water) and the mean measured bulk relative permittivities ($\bar{\varepsilon}_{r-bulk}$) of MeOH:1PrOH mixtures with permittivity values as close as possible to the targeted glycine (gly.) solutions shown in* ***Table S1****. The mean was obtained from "n" repeat measurements. Also shown are the MeOH:1PrOH volume percentages used to achieve this "index matching", and the percentage differences (diff.%) of $\bar{\varepsilon}_{r-bulk}$ from literature and corresponding glycine solution values.*

n	Target conc. (g/kg)	Solvent system	MeOH vol.%	1PrOH vol.%	ε_{r-bulk} literature value[a,b]	$\bar{\varepsilon}_{\text{r-bulk}}$ [c,d] measured	SD[e] of measure -ments	Diff.% from lit. value	Diff.% from gly. sol.[e]
9	na	Methanol (MeOH)	na	na	1.7609	1.7611	0.0008	0.01%	na
22	0	Water	na	na	1.7737	1.7732	0.0001	-0.03%	na
9	50	MeOH:1PrOH	74.0%	26.1%	na	1.8020	0.0053	na	0.30%
9	100	MeOH:1PrOH	61.0%	39.1%	na	1.8152	0.0036	na	-0.13%
9	125	MeOH:1PrOH	55.4%	44.6%	na	1.8261	0.0012	na	-0.05%
10	150	MeOH:1PrOH	47.6%	52.4%	na	1.8338	0.0045	na	-0.18%
26	176	Ethanol	na	na	1.8507	1.8481	0.0013	-0.14%	0.07%
11	200	MeOH:1PrOH	34.5%	65.6%	na	1.8574	0.0023	na	0.10%
9	250	MeOH:1PrOH	23.7%	76.4%	na	1.8711	0.0025	na	-0.06%
6	na	1-Propanol (1PrOH)	na	na	1.9121	1.9122	0.0005	0.00%	na

[a] See **Table S3** for literature sources of the listed values; [b] The refractive index is square root of ε_r; [c] A stroke on top of the symbol for relative permittivity (ε_r) denotes the mean; [d] SD denotes standard deviation; [e] Difference from values shown in **Table S1**.

Table S3. *Literature values of refractive index (n) and calculated ε_{r-bulk} = n^2 for water, methanol, ethanol, and 1-propanol. In black are reference values with measurement wavelength and temperatures (632.8 nm, 21.5-22ºC) corresponding to the present study (632.8 nm, 21.4ºC - 22.3ºC). For comparison, in gray are literature values with either wavelength or temperature not matching the present study.*

Solvent	Reported $\varepsilon_r = n_r^2$	Reported nr	Wavelength used in study	Temperature used in study	Source
Water	1.7732	1.3316	632.8 nm	22ºC	Tilton & Taylor, 1938 (interpol.)[a]
Water	1.7742[a]	1.3320[a]	632.8 nm	21.5ºC	Daimon et al., 2007 (interpol.)[a]
Water	1.7714790	1.3309692	656.28 nm	22ºC	Tilton & Taylor, 1938 [1]
Water	1.7763649	1.3328034	589.26 nm	22ºC	Tilton & Taylor, 1938 [1]
Water	1.773369	1.331679	644.025 nm	21.5ºC	Daimon et al., 2007 [2]
Water	1.777582	1.333260	587.825 nm	21.5ºC	Daimon et al., 2007 [2]
Methanol	1.7609	1.327	632.8 nm	21.5ºC	Chang et al., 2004 [3]
Methanol	1.759186	1.326343	632.8 nm	25ºC	El-Kashef et al., 2000 [4]
Methanol	1.7609	1.3270	632.8 nm	25ºC	Kozma et al, 2004 [5]
Ethanol	1.8507	1.3604	632.8 nm	21.6ºC	Chang et al., 2004 [3]
Ethanol	1.8507	1.3604164	632.8 nm	20ºC	Rheims et al., 1997 [6]
Ethanol	1.8477	1.3593	632.8 nm	25ºC	Kozma et al, 2004 [5]
1-Propanol	1.9121	1.3828	632.8 nm	21.7ºC	Chang et al., 2004 [3]
1-Propanol	1.9129[a]	1.3831[a]	632.8 nm	22ºC	O'Brien et al, 1968 (interpol.)[a]
1-Propanol	1.91496	1.38382	632.8 nm	20ºC	O'Brien et al, 1968 [7]
1-Propanol	1.90981	1.38196	632.8 nm	25ºC	O'Brien et al, 1968 [7]

[a] Interpolated values based on data from a single study/publication with either measurement wavelengths or temperatures, but not both, bracketing the present experimental conditions.

Table S4. *The thickness and relative permittivity (ε_r) values of Cr and Au layers. There were three batches of SPR chips corresponding to metal layers deposited at different times. As expected in vapor deposition, the values within each batch closely matched with each other but the batch averages can vary. Matching of SPR data with Cr and Au parameters were significantly aided by the semi-independent ways that different parameters modify the reflectivity data: i) the Cr and Au thickness values have dominant control over the slopes after and before the θ_{TIR} angle, respectively; ii) the Cr layer is comparatively so much thinner than Au that variations in ε_{r-Cr} have little influence and a standard value consistent with the literature and past samples was used; and iii) the real and imaginary parts of ε_{r-Au} control the θ_{SPR} angle position and the width of the reflectivity minimum centered around θ_{SPR}. The (complex) n_r of the average Cr and Au permittivities are also shown and are similar to literature values [8]. The standard deviation (SD) of ε_{r-Au} refer to the small variations (0.5-3%) between measurements using the same SPR chip (see **Table S1** and **Table S2**).*

	Chip number	Cr thickness (nm)	$\varepsilon_{r-\mathrm{Cr}}$		Au thickness (nm)	$\varepsilon_{r-\mathrm{Au}}$	SD $\varepsilon_{r-\mathrm{Au}}$ (% magnitude)
**Batch 1**	Au-11	0.1	-1.15+19i		46	-10.3+1.6i	0.7%
	Au+PS-4	0.1	-1.15+19i		46	-10.7+1.8i	1.9%
	**Average**	**0.1**	**-1.15+19i**		**46**	**-10.5+1.7i**	
			(n = 2.99+3.18i)			(n = 0.26+3.25i)	
**Batch 2**	Au-1	0.1	-1.15+19i		46	-12.4+1.6i	0.7%
	Au-2	0.1	-1.15+19i		46	-12.2+1.7i	0.7%
	Au-3	0.1	-1.15+19i		46	-12.2+1.7i	0.8%
	Au-4	0.2	-1.15+19i		45	-12.3+1.7i	0.6%
	Au-5	0.1	-1.15+19i		45	-12.3+1.7i	0.5%
	Au-6	0.1	-1.15+19i		46	-12.3+1.4i	0.7%
	Au-7	0.1	-1.15+19i		46	-12.2+1.7i	2.7%
	Au-8	0.1	-1.15+19i		46	-12.1+1.7i	2.4%
	Au-9	0.1	-1.15+19i		48	-11.5+1.7i	1.1%
	Au-10	0.1	-1.15+19i		46	-12.4+1.6i	0.6%
	Au+PS-1	0.1	-1.15+19i		46	-12.0+1.8i	1.9%
	Au+PS-2	0.1	-1.15+19i		46	-12.9+2.0i	1.5%
	Au+PS-3	0.1	-1.15+19i		46	-12.6+1.7i	1.4%
	**Average**	**0.1**	**-1.15+19i**		**46**	**-12.8+1.8i**	
			(n = 2.99+3.18i)			(n = 0.26+3.58i)	
**Batch 3**	Au+PS-5	3.2	-1.15+19i		59	-13.0+1.5i	3.2%
	Au+PS-6	3.3	-1.15+19i		57	-12.9+1.4i	2.2%
	**Average**	**3.3**	**-1.15+19i**		**58**	**-12.9+1.5i**	
			(n = 2.99+3.18i)			(n = 0.20+3.60i)	

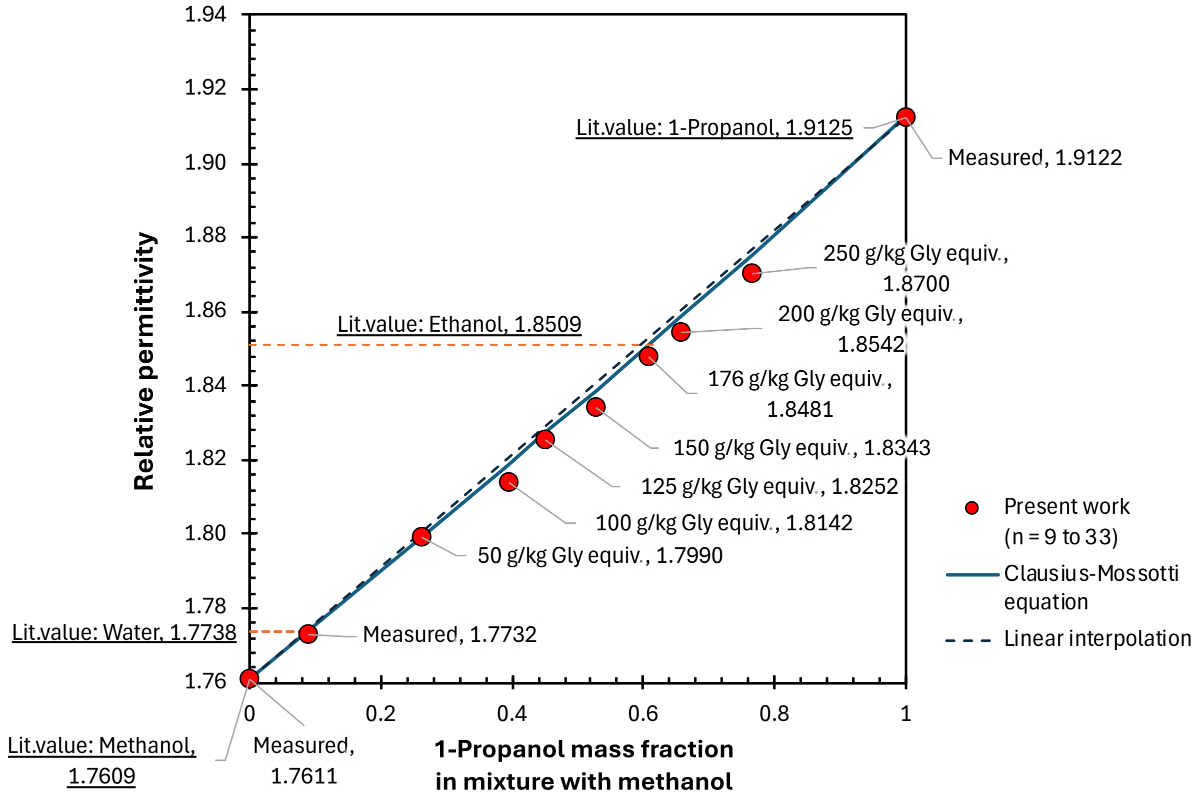

Figure S1. *Comparison of measured bulk permittivities of both pure solvents and index-matched methanol:1-propanol (MeOH:1PrOH) mixtures with literature values, the values obtained from the Clausius-Mossotti equation [9] (see Section S4) and the values from an arithmetic interpolation of the pure MeOH and 1PrOH solvents. The temperature recorded during the measurements was between 21.4-22.3 ºC (mean = 21.8 ± 0.24) and the measurements were based on the critical total internal reflection angle of a 632.8 nm HeNe laser. See* ***Table S2*** *for the plotted values and standard deviations at each condition. See* ***Table S3*** *for literature reference values. The Clausius-Mossotti equation predicts ε_{r-bulk} values closer to the experimental values than a simple linear interpolation, although the values are still slightly too high due to the assumption of ideal molecular mixing. Nonetheless, the differences are <0.3%.*

Table S5. *SPR measurement data values for the "thiol" (dodecanethiol SAM) and PS (spin-coated polystyrene) layers used for PS-glycine solution interface measurements. See **Table S4** for details on batch numbering and the Cr and Au parameter values for each SPR chip.*

	Chip number	Thiol thickness (nm)	PS thickness (nm)
**Batch 1**	Au+PS-4	1.2	8.0
**Batch 2**	Au+PS-1	2.8	7.3
	Au+PS-2	2.6	8.7
	Au+PS-3	1.6	8.7
**Batch 3**	Au+PS-5	1.6	15.3
	Au+PS-6	1.6	14.5

S2. Measurement data of glycine solutions on Au and PS sensor chips

Table S6. *SPR measurement data values for the Au-glycine solution interface. The mean concentrations, relative permittivity of the bulk liquid ($\bar{\varepsilon}_{r-bulk}$) and of a 1 nm interfacial region ($\bar{\varepsilon}_{r-interf}$) were measured across "n" repeated number of measurements. SD denotes standard deviation; SEM denotes standard error of the mean, i.e., the expected bounds of the average given the number of measurements, calculated as SEM = SD/√n. The ±1 SEM is shown as error bounds in the main text and accompanying Figure 5a.*

n (no. of repeats)	Concentration (average of repeats) (g/kg)	SD of concentration (g/kg)	$\bar{\varepsilon}_{r\text{-bulk}}$	SD of $\bar{\varepsilon}_{r\text{-bulk}}$	$\bar{\varepsilon}_{r\text{-interf}}$	SD of $\bar{\varepsilon}_{r\text{-interf}}$	SEM of $\bar{\varepsilon}_{r\text{-interf}}$
10	0	na	1.7732	0.0001	1.7732	0.0001	0.0000
9	49.987	0.009	1.7965	0.0013	1.8432	0.0741	0.0247
9	99.967	0.017	1.8177	0.0007	1.9331	0.1123	0.0374
9	125.074	0.053	1.8272	0.0004	1.9515	0.0883	0.0294
9	149.945	0.036	1.8373	0.0007	1.9385	0.1329	0.0443
18	175.977	0.000	1.8467	0.0003	2.0717	0.1417	0.0334
13	199.993	0.049	1.8555	0.0009	2.0770	0.1759	0.0488
9	249.958	0.070	1.8728	0.0009	1.9997	0.1073	0.0358

Table S7. *SPR measurement data values for the PS-glycine solution interface. The mean concentrations, relative permittivity of the bulk liquid ($\bar{\varepsilon}_{r-bulk}$) and of a 1 nm interfacial region ($\bar{\varepsilon}_{r-interf}$) were measured across "n" repeated number of measurements. SD denotes standard deviation; SEM denotes standard error of the mean, i.e., the expected bounds of the average given the number of measurements, calculated as SEM = SD/√n. The ±1 SEM is shown as error bounds in the main text and accompanying Figure 5b.*

n (no. of repeats)	Concentration (average of repeats) (g/kg)	SD of concentration (g/kg)	$\bar{\varepsilon}_{r\text{-bulk}}$	SD of $\bar{\varepsilon}_{r\text{-bulk}}$	$\bar{\varepsilon}_{r\text{-interf}}$	SD of $\bar{\varepsilon}_{r\text{-interf}}$	SEM of $\bar{\varepsilon}_{r\text{-interf}}$
12	0	na	1.7732	0.0001	1.7732	0.0001	0.0000
11	50.003	0.023	1.7966	0.0008	2.0256	0.1801	0.0543
12	99.967	0.048	1.8174	0.0005	2.0341	0.1475	0.0426
11	125.043	0.047	1.8269	0.0013	2.1110	0.1387	0.0418
12	149.992	0.055	1.8368	0.0009	2.0762	0.1110	0.0320
12	175.892	0.060	1.8468	0.0008	2.0079	0.0927	0.0268
10	200.010	0.133	1.8555	0.0007	2.0266	0.0761	0.0241
11	250.015	0.115	1.8717	0.0020	2.0452	0.1037	0.0313

S3. Permittivities measured for individual sample chips

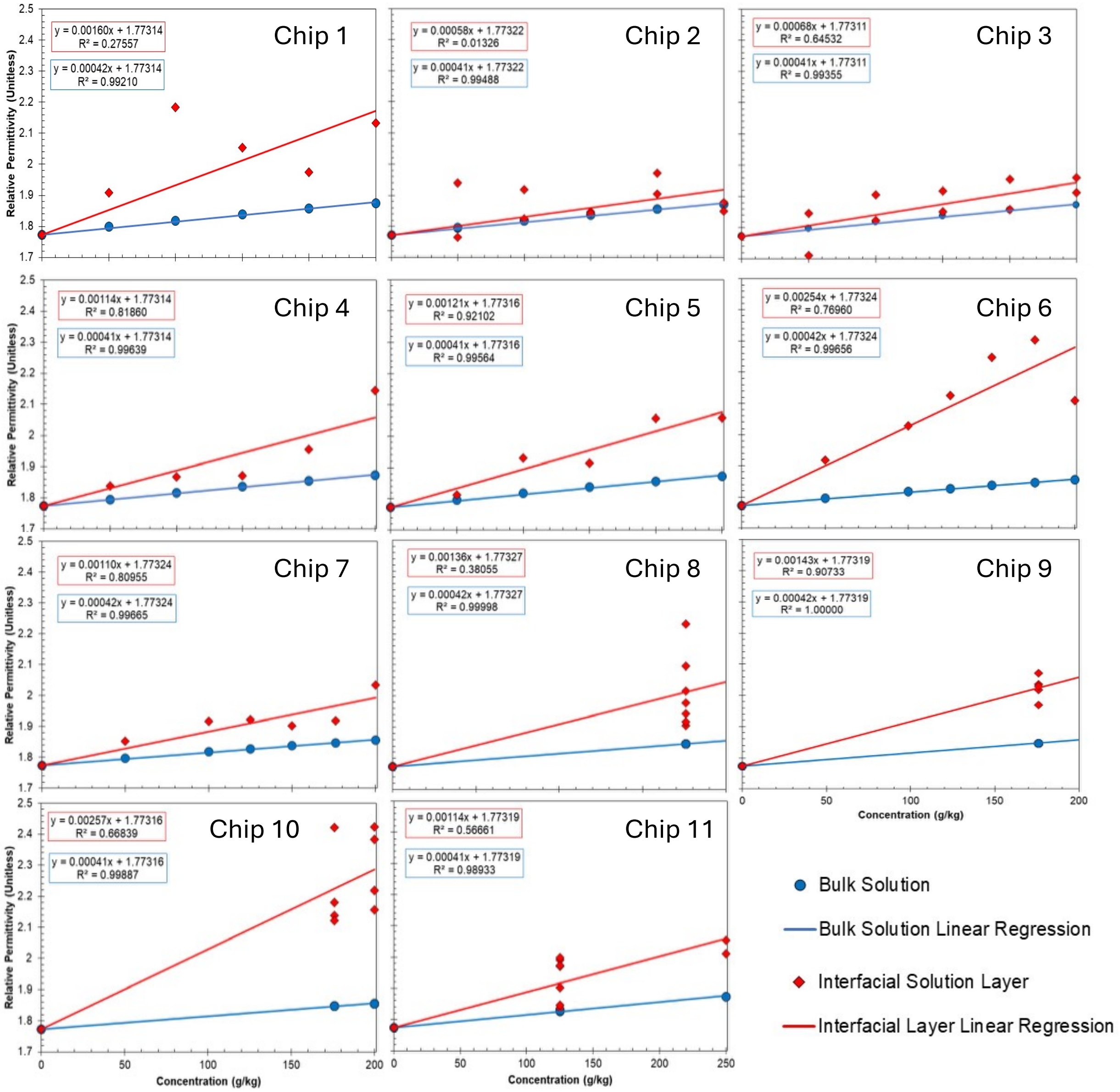

Figure S2. *Plots of* $\bar{\varepsilon}_{r-interf}$ *and* $\bar{\varepsilon}_{r-bulk}$ *measured at different bulk glycine concentrations on each individual gold coated SPR sensor chips (Au chips). The temperature recorded during the measurements was between 21.5-22.3ºC. See* ***Table S1*** *for* $\bar{\varepsilon}_{r-bulk}$ *values and* ***Table S6*** *for mean values and the standard deviations and standard errors of* $\bar{\varepsilon}_{r-interf}$*. See* ***Table S4*** *for the thickness and permittivity values of Cr and Au for each SPR chip.*

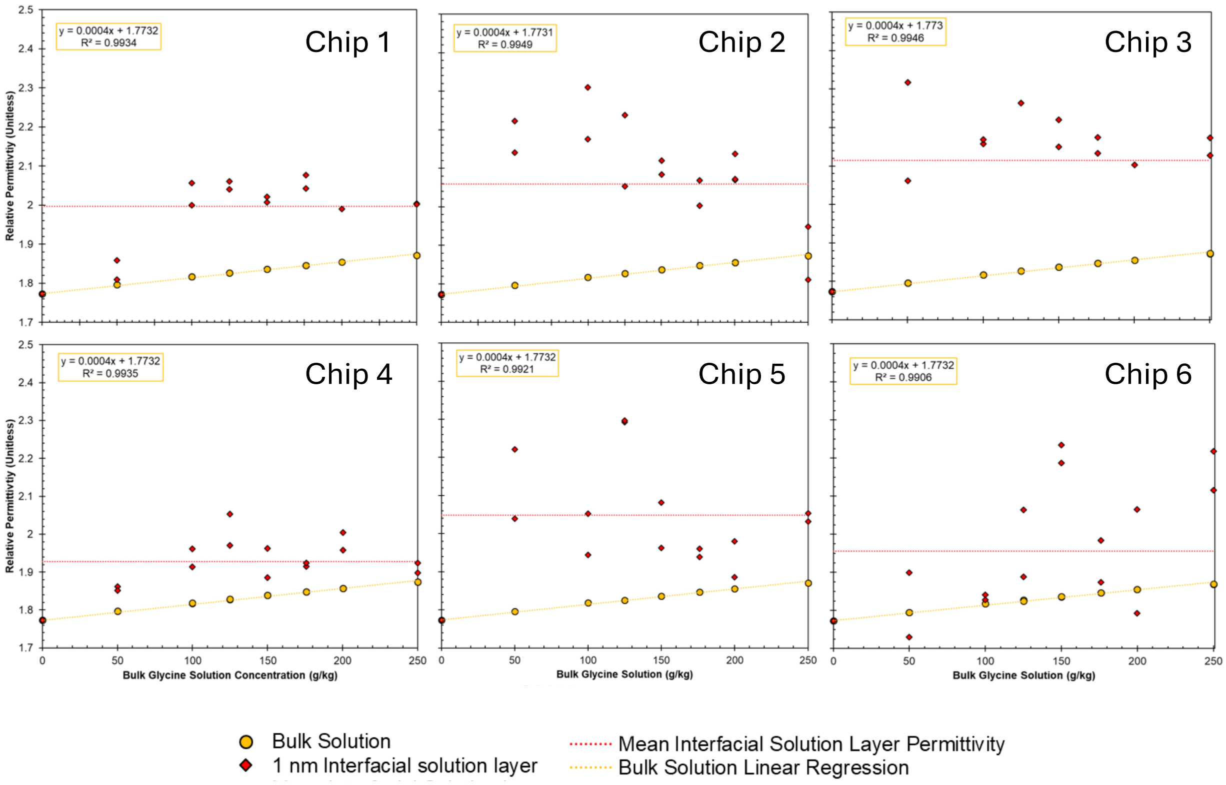

Figure S3. *Plots of* $\bar{\varepsilon}_{r-interf}$ *and* $\bar{\varepsilon}_{r-bulk}$ *measured at different bulk glycine concentrations on each individual gold coated SPR sensor chips (Au+PS chips). The temperatures recorded during the measurements was between 21.4-22.3ºC. See* ***Table S1*** *for* $\bar{\varepsilon}_{r-bulk}$ *values and* ***Table S7*** *for mean values and the standard deviations and standard errors of* $\bar{\varepsilon}_{r-interf}$*. See* ***Table S4*** *for the thickness and permittivity values of Cr and Au, and* ***Table S5*** *for the permittivity values of thiol and PS, for each SPR chip.*

S4. Estimating ε_r of mixtures based on the Clausius-Mossotti equation

The Clausius-Mossotti equation is a basic description in electromagnetism relating atomic-scale polarizability and the bulk material property of relative permittivity (ε_r), assuming homogeneous ideal mixing [9]. For non-magnetic materials, $\varepsilon_r = n^2$, the square of refractive index (*n*). For a mixture of multiple molecular species, the equation can be written in the form:

$$\frac{\varepsilon_r - 1}{\varepsilon_r + 2} = \sum_i \frac{N_i \alpha_i}{3\varepsilon_0}$$

Equation 1

where N_i is the number density of molecular species i, α_i is the polarizability of the species i, and ε_0 is the permittivity of free space. The equation as shown applies when the frequency of the electromagnetic (EM) waves concerned is high enough (e.g., high terahertz visible light frequencies, as with the 632.8 nm red laser used in the present SPR setup) such that physical molecular motions giving rise to permanent dipole moment effects are too slow to follow the EM wave oscillation. Otherwise, a dipole moment term can be added within the summation.

To calculate ε_r of mixtures, e.g., methanol and 1-propanol, and water and glycine, α_i of each single component material *i* has to first be obtained. For single components, Equation 1 can be simplified to:

$$\alpha = \frac{3\varepsilon_0}{N} \frac{\varepsilon_r - 1}{\varepsilon_r + 2}$$

Equation 2

Values of ε_r were obtained from literature reports of the refractive index ($\varepsilon_r = n^2$) and N can be calculated from the mass density (ρ) and molar mass (M) ($N = N_A \rho M$, where N_A is Avogadro's number). For literature ε_r and ρ values see **Table S3**, **Table S8** to **Table S11**. A value of 0.9976 g/mL for water at the experimental temperature of 22ºC published by the US National Institute of Standards and Technology was used).[10] The values of α_i calculated for each single component material is given in **Table S11**.

By relating the concentration of glycine to the corresponding volumetric densities and numbers of glycine and water molecules in a standard volume, the experimental interfacial $\bar{\varepsilon}_{r\text{-interf}}$ can be related to glycine mass fraction (x_{gly}) through Equations 1, as shown in **Figure S4**a. This shows that the range of $\varepsilon_{r\text{-interf}}$ measured (up to ~2.1) implies glycine mass fractions (x_{gly}) of up to ~0.6. Also plotted in the same figure are the experimental values of the present study and the aforementioned literature reference values. The correspondence between bulk glycine concentration and mass fraction is given in **Table S12**.

An average of the glycine crystal permittivities reported for different polymorphs was used in calculations with the Clausius-Mossotti equation (see **Table S9** for individual values), to not prejudice the estimation of x_{gly} based on any particular crystalline form for fully dense glycine. Moreover, the lower $\varepsilon_{r\text{-bulk}}$ values of the crystals reported were deemed unphysical because the highest measured values of $\varepsilon_{r\text{-interf}}$ would imply very large x_{gly} approaching unity (i.e., fully dense layers, which were not observed). The higher values of $\varepsilon_{r\text{-bulk}}$ were also considered to be less

applicable because they would imply very high non-linearity in the x_{gly} range > 0.2 in order to account for the values of bulk glycine solutions known from both the literature and the present study. Hence, the average value of of $\varepsilon_{r\text{-bulk}}$ = 2.379 at x_{gly} = 1 appears to be a reasonable choice.

Regardless of the polymorph, the use of the Clausius-Mossotti equation to estimate x_{gly} from $\varepsilon_{r\text{-interf}}$ is justified by i) the fundamental applicability of the equation, ii) the excellent agreement between literature data and the measured $\varepsilon_{r\text{-bulk}}$ of glycine solutions available in the range of x_{gly} up to 0.2 (a slightly depressed permittivity for a given x_{gly} compared to a linear interpolation between the pure phases is correctly predicted), and iii) by the fact that the range of interfacial $\varepsilon_{r\text{-interf}}$ measured at 1.9 ~ 2.1 lies mid-way between the values for lower concentration solutions and fully dense glycine (i.e., crystals), and hence x_{gly} at the interface must also lie between those for the bulk solutions and the crystals. For any reasonable value of permittivity, the measured values of interfacial $\varepsilon_{r\text{-interf}}$ would give x_{gly} estimates up to ca. 0.6~0.7 as shown.

Table S8. *Literature values of mass density (ρ) for various glycine crystals. The average value was used for calculation of the volumetric number density, N_{gly} (see Section S0 for details).*

Polymorph	ρ (g/mL)	Source
na	1.6	[11]
na	1.623	[11]
na	1.5767	[11]
na	1.574	[11]
δ	1.76	[12]
α	1.608	[12]
β	1.577	[12]
Average	1.6169	

Table S9. *Literature values of refractive index (n) and the calculated bulk relative permittivity ($\varepsilon_{r\text{-bulk}}$) for various glycine crystals. The average value was used for calculation of α_{gly}. The permittivity is related to the refractive index by $\varepsilon_{bulk} = n^2$.*

Polymorph	n	$\varepsilon_{r\text{-bulk}}$	Source
α	1.459	2.13	[13]
α	1.584	2.51	[14]
β	1.578	2.49	[14]
γ	1.58	2.5	[14]
γ	1.520	2.31	[15]
γ	1.616	2.61	[16]
γ	1.45	2.1	[17]
Average		2.379	

Table S10. *Literature values of the volumetric mass density (ρ) for methanol and 1-propanol. Data for a temperature matching the present measurement (22°C, see Figure S1 caption) was not found. As an approximation, literature densities from 16.85^{o}C , 20°C , 25°C and 26.85^{o}C were interpolated, and these were averaged to obtain an estimate corresponding to 22°C.*

Substance	ρ (g/mL)	Temperature (^{o}C)	Literature source
Methanol	0.7943	16.85	Goodwin, et al., 1987[18]
Methanol	0.79191	20	Hevia et al., 2022 [20]
Methanol	0.7914	20	Ganz et al., 1972 [19]
Methanol	0.7900	22	Interpolated from Hevia et al., 2022[a]
Methanol	0.7898	22	Average of interpolations used in this study
Methanol	0.7896	22	Interpolated from Goodwin, et al., 1987 [a]
Methanol	0.7872	25	Hevia et al., 2022 [20]
Methanol	0.7849	26.85	Goodwin, et al., 1987[18]
1-propanol	0.80361	20	Hales et al., 1976 [21]
1-propanol	0.80352	20	Hevia et al., 2022 [20]
1-propanol	0.80201	22	Interpolated from Hales et al., 1976 [a]
1-propanol	0.8020	22	Average of interpolations used in this study
1-propanol	0.80192	22	Interpolated from Hevia et al., 2022 [a]
1-propanol	0.7996	25	Hales et al., 1976 [21]
1-propanol	0.79951	25	Hevia et al., 2022 [20]

[a] Linear interpolation based on data from a single study/publication with temperatures bracketing the present experimental conditions.

Table S11. *Values of molecular polarizability (α) at 22°C based on the Clausius-Mossotti equation and corresponding literature values of the volumetric mass density (ρ), number density (N), and relative permittivity (ε_r). See Section S4, ref.10, **Table S8** and **Table S10** for details.*

Component	Molecular weight (g/mol)	Mass density, ρ (g/mL)	Number density, *N* (no./mol/m^3)	Relative Permittivity ε_r	Polarizability, α (Å^{2}s^4/kg)
Water	18.018	0.9976	3.3326×10^{28}	1.773	1.6332×10^{-40}
Glycine	75.07	1.6169	1.2971×10^{28}	2.379	6.4475×10^{-40}
Methanol	32.042	0.7898	1.4833×10^{28}	1.761	3.6231×10^{-40}
1-Propanol	60.096	0.8020	8.0323×10^{27}	1.912	7.7104×10^{-40}

Table S12. *Correspondence between bulk glycine solution concentration (glycine mass/1 kg water) and the glycine mass fraction (x_{gly} = glycine mass/(glycine mass + water mass)) for the concentration values used in this study. The corresponding volumetric mass density, estimated from fitting (**Figure S4**) of literature values [22], is also shown.*

Bulk concentration (g/kg)	Bulk mass fraction (x_{gly})	Volumetric mass density (g/cm^3)
0	0.000	0.9976
50	0.048	1.0167
100	0.091	1.0350
125	0.111	1.0438
150	0.130	1.0524
176	0.150	1.0612
200	0.167	1.0690
250	0.200	1.0848

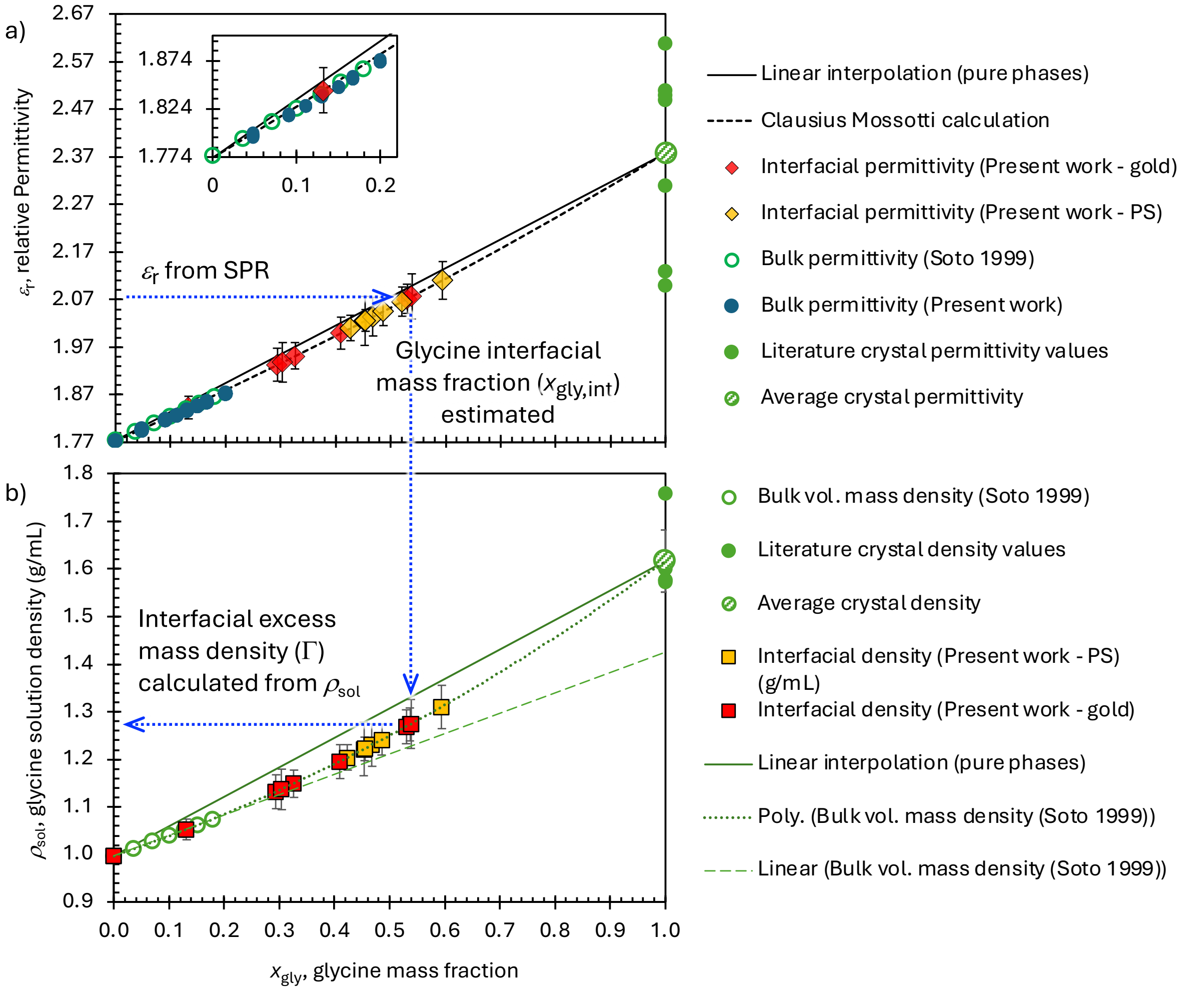

Figure S4 *a) Glycine mass fraction (x_{gly}) is estimated from the measured bulk $\varepsilon_{r\text{-}bulk}$ and interfacial $\varepsilon_{r\text{-}interf}$ (see **Table S1, Table S6** and **Table S7**, respectively, for values) through the Clausius-Mossotti equation (dashed line; see Section S4 and Equation 1 for details). The plot also compares these values with literature relative permittivity data of glycine solutions (Soto 1999 [22]). A linear interpolation (solid line) between literature $\varepsilon_{r\text{-}bulk}$ values of water (at x_{gly} = 0) and different glycine crystal polymorphs (x_{gly} = 1) is also shown for comparison (see **Table S3** and **Table S9** for values and references). b) The volumetric mass density (ρ) is related to x_{gly} through a polynomial interpolation (dotted line; $\rho = 0.2288\, x_{gly}^2 + 0.3905\, x_{gly} + 0.9976$) of literature data available for glycine solutions (Soto 1999 [22]) and for glycine crystal polymorphs (**Table S8**). In comparison, linear interpolations based on either fitting the literature glycine solution values (dahed green line) or the x_{gly} = 0 and 1 end point values for the density of water (**Table S11**) and the average of glycine polymorphs (solid green line) fail to account for all the literature values.*

S5. Estimating excess interfacial mass density (Γ)

The excess interfacial mass density Γ of glycine is defined as the mass density of glycine in the interfacial layer minus the mass density of glycine in the bulk solution per unit area of the interface.

In conventional SPR applications, Γ is either estimated based on calibrated "reflectivity units" or the refractive index of the molecule, hence $\varepsilon_{\text{r-interf}}$, is separately measured in bulk samples so that thickness can be determined from θ_{SPR} shifts—an overestimation of $\varepsilon_{\text{r-interf}}$ would result in underestimation of the thickness, and *vice versa*. However, in the present study, the presence of $\varepsilon_{\text{r-interf}}$ is solely an interfacial phenomenon and cannot be determined independently.

In the present SPR measurements, the relative permittivity of the interphase region, $\varepsilon_{\text{r-interf}}$, is obtained from the coupling angle using the WINDSPALL software while assuming an interfacial layer thickness of $t = 1$ nm. From the value of $\varepsilon_{\text{r-interf}}$, the corresponding solution concentration, expressed as mass fraction of glycine, x_{gly} can be estimated using the Clausius-Mossotti equation, as described in Section 4 (**Figure S4a**).

The solution volumetric density ρ corresponding to a given mass fraction of glycine is obtained from the relationship between ρ and x_{gly}, which was obtained by fitting literature data for pure water ($x_{gly} = 0$) and glycine crystals ($x_{gly} = 1$) (**Figure S4b** and **Table S11**) as well as for bulk glycine solutions measured at several concentrations (values of ρ available for $x_{gly} = 0.04$ to 0.18 from ref.[22]). **Figure S4b** shows that a polynomial fit can describe all these literature values while a linear fit can only describe either the literature glycine solution values or the pure water and glycine crystal end point values. Based on the polynomial fit, values of bulk solution density ρ_{bulk} were calculated from $x_{gly,bulk}$ for the various bulk concentrations used in the present study (**Table S12**). Given the values of $x_{gly,int}$ in the interfacial solution layer estimated using the Clausius-Mossotti equation (see blue arrows indicated in **Figure S4**), the solution volumetric mass density $\rho_{sol,int}$ at the interface was also calculated based on the aforementioned polynomial fit.

We can then estimate the mass of glycine in the interfacial layer across a surface area *A* (e.g., 1 cm^2). For t = 1 nm = 10^{-7} cm, The mass of glycine in a volume $V = A \times t = 10^{-7}$ cm^3 in the interfacial region is equal to the solution density ($\rho_{sol,int}$) multiplied by the corresponding mass fraction of glycine ($x_{gly,int}$) multiplied by the volume V so:

$$m_{gly,int} = \rho_{sol,int}\, x_{gly,int}\, V$$

We can work out the mass in a bulk solution of equivalent volume using:

$$m_{gly,bulk} = \rho_{sol,bulk}\, x_{gly,bulk}\, V$$

Finally, the excess mass per surface area Γ can be calculated using:

$$\Gamma = \frac{m_{gly,int} - m_{gly,bulk}}{A}$$

However, it should be noted that the analysis can begin with any assumed value of t—a larger t results in a lower $\varepsilon_{\text{r-interf}}$ to account for the same magnitude of SPR angle shift measured, and vice versa. Γ is therefore invariant with t.

For example, assuming t = 1 nm, analysis of SPR data measured for the 176 g/kg bulk glycine solution on a Au surface gave $\varepsilon_{r\text{-interf}}$ = 2.0717 and $\varepsilon_{r\text{-bulk}}$ = 1.8467 (**Table S6**). **Figure S4a** shows that the corresponding glycine mass fractions at the interface and in the bulk are $x_{gly,int}$ = 0.530 and $x_{gly,bulk}$ = 0.150, respectively. Accordingly, **Figure S4b** shows that the densities of the glycine solutions at the interface and in the bulk are $\rho_{sol,int}$ 1.2691 g/cm^3 and $\rho_{sol,bulk}$ 1.0612 g/cm^3, respectively. The mass per volume of the glycine content within these solution environments are therefore $m_{gly,int} = \rho_{sol,int} \times x_{gly,int}$ = 0.673 g/cm^3 and $m_{gly,bulk} = \rho_{sol,bulk} \times x_{gly,bulk}$ = 0.159 g/cm^3. The excess mass of glycine at the interface is simply the difference between the interfacial and bulk background values = 0.673 – 0.159 = 0.514 g/cm^3.

For a unit area of 1 cm^2 and t = 1 nm, this volumetric excess is equivalent to an excess surface mass density of Γ = 51.4 ng/cm^2 (see **Figure S5** for illustration of the conversion). The calculated values of Γ for the full set of $\varepsilon_{r\text{-interf}}$ and $\varepsilon_{r\text{-bulk}}$ data measured on at the Au and PS surfaces (**Table S6** and **Table S7**) are plotted in **Figure 3** panels c and d in the main text.

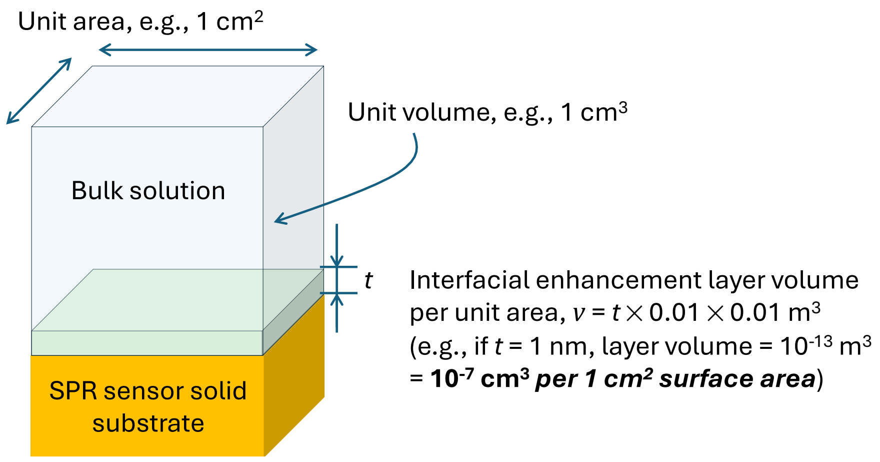

Figure S5. Schematic illustrating the dimensions of the interfacial layer with enhanced glycine concentration with thickness t and a 1 cm^2 unit area.

The main variables in the above estimation of Γ are the values of $\varepsilon_{r\text{-bulk}}$ and ρ for crystalline glycine used, respectively, in the estimations of x_{gly} and ρ of the interfacial layer. Literature values for different glycine crystalline polymorphs can differ significantly (see **Table S8** and **Table S9**). In general, average values were used to not prejudice the estimation based on any particular crystalline form, and for $\varepsilon_{r\text{-bulk}}$, the use of the averaged crystalline glycine value reported is already justified in Section 4. For ρ, the averaged value of different polymorphs = 1.617 g/cm^3 used (see **Table S8**) is justified by the fact that the range of densities reported is relatively small (1.57 g/cm^3 to 1.62 g/cm^3, except for one value of 1.76 reported for δ-glycine) and the estimation of Γ should not differ drastically upon changes to the density value used for the calculation.

S6. Detailed Experimental Methods

S6.1. Materials and Labware Cleaning

Methanol (99%, VWR), ethanol (>99.5% non-denatured, Fisher Scientific), 1-propanol (99.9% HPLC grade, Sigma Aldrich), 1-dodecanethiol (≥98%, Sigma Aldrich), polystyrene (PS) (Mw 34,300 g mol^{-1}, Polymer Source Inc., Dorval, Canada), toluene (laboratory reagent, ≥99.3%, Sigma Aldrich), glycine (98.5%, Fisher Scientific) and Hellma™ Hellmanex Cleaning Solution (Fisher Scientific) were purchased and used as received. All glass and other labware used were cleaned using a Hellmanex solution (10 min sonication in 1% Hellmanex in deionised (DI) water, followed by 10-times exchange/rinsing in DI water, 10 min sonication in DI water, and final exchange/rinsing in ethanol before drying under a stream of nitrogen gas).

S6.2. Gold (Au) Chip and Polystyrene (PS) Coating Preparation

All SPR sensor chips used double-sided, precision ground glass slide substrates (1.5 mm x 20 mm × 20 mm pieces, quarter wavelength flatness, ChangChun Long Ze Precision Optics Co. Ltd., China) with refractive index (n_r) matching the LaSFN9 glass prism. All chips were first deposited with a thin chromium (Cr) adhesion layer followed by a gold (Au) layer, using a Q150R S sputter coater (Quorum Technologies). Visual inspection by optical microscopy (Nikon Eclipse LV100) was performed on samplings of SPR chip batches (in brightfield to check for gross defects and in darkfield to reveal scattering from nanoscopic defects). Samples with noticeable surface defects were rejected. All chips were cleaved into ~1x1 cm^2 pieces before use. For preparing PS coated Au SPR chips, the original Au surface was first functionalized with a hydrophobic 1-dodecanethiol self-assembled monolayer (SAM) to promote PS wetting (3-times 30 s oxygen plasma treatment followed by incubation in 1 mM 1-dodecanethiol in ethanol for 1 hour, rinsing with fresh ethanol, then water, and dried under stream of nitrogen). Then, a 0.3 w/v% solution of PS dissolved in toluene was spin-coated onto the Au chips (50 µL PS solution pipetted onto the Au chip, then 1500 rpm 60 seconds using a Chemat Technology KW-4A unit) to deposit ~10 nm coatings. These samples were dried for 24 hours in air and all chips were examined under an optical microscope (Leica M165 CM) to screen out chips with de-wetting defects before use. Samples with noticeable surface defects were rejected. The thickness values of the metal and organic coating layers for each individual chip were measured by SPR (see Section 6.6).

S6.3. Solution Preparation for SPR Measurements

Aqueous glycine solutions at concentrations of 50, 100, 125, 150, 176, 200 and 250 $g_{glycine}/kg_{water}$ (denoted as g/kg hereafter) were prepared for SPR measurements. Glycine powders were weighed, mixed in DI water in glass vials, and sonicated for up to 10 minutes. Dissolution was confirmed by visual inspection. The solutions were further left for at least 1 hour before measurements to equilibrate to room temperature and to further ensure dissolution. Binary mixtures of methanol and 1-propanol were also prepared to provide solvent solutions with n_r matching those of the 50, 100, 125, 150, 200 and 250 g/kg glycine solutions. Ethanol was used to match the n_r of 176 g/kg glycine solution.

Table S1 shows that the glycine solutions were prepared to within 0.05% of target concentrations and that the bulk n_r of these solutions had standard deviations within 0.1% of the average values (measured from SPR reflectivity scans, see Section 6.5). **Table S2** further shows the mixing ratios of methanol and 1-propanol of the index matched solutions, as well as the comparisons of n_r

between the glycine solutions and literature values of water, methanol, ethanol and 1-propanol. See **Table S3** for literature reference values and also **Figure S1** displaying the comparisons.

S6.4. SPR Spectrometer Setup

A purpose built SPR setup in the Kretschmann configuration [23] based on an established design with a 90º high index LaSFN9 glass prism [24] (**Figure S6**) was used to acquire reflectivity angle scan spectra. A monochromatic 632.8 nm laser (He/Ne, R-30989, Research Electro Optics Inc, Colorado, USA) was directed through an optical chopper connected to a lock-in amplifier (SR540 and SR350, respectively, from Stanford Research Systems, California, USA) and a pair of linear polarisers (Thorlabs Inc., New Jersey, USA). The laser intensity was adjusted using the polarisers and incidence polarization set to transmagnetic with respect to the SPR sensor surface. The polarized light was directed into and reflected off the internal "back-side" major flat surface of the SPR prism-Au sensor chip assembly joined together with index matching fluid (Cargille Laboratories, New Jersey, USA). The reflected intensity was focused by two convex N-BK7 glass lenses onto a silicon biased photodetector (DET100A2 fitted with a 50 Ω BNC terminator, Thorlabs). A custom-made polytetrafluoroethylene (PTFE) liquid cell with an internal volume of 270 µL and inlet and outlet injection ports was placed on the chip and sealed by perfluoroelastomer O-rings and a glass slide back-cover. The entire prism, SPR chip and liquid cell assembly was secured by a plastic cover piece in a 3D printed holder mounted on a rotation stage (HDR50/M, Thorlabs, New Jersey, USA). Stage control and SPR reflectivity data acquisition were performed through a user interface set up on the LabVIEW software platform. Temperature was separately monitored by HH309A Data logger thermometers (Omega Engineering, Manchester, U.K.), with one probe monitoring laboratory temperature and another monitoring the liquid cell. The temperature during measurements was between 22±0.23ºC (range: 21.4ºC to 22.3ºC).

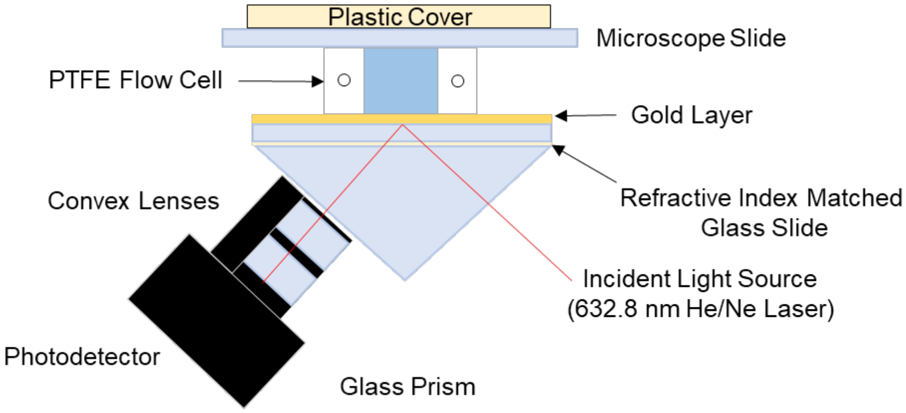

Figure S6. *Diagram showing key components of the SPR spectrometer setup.*

S6.5. SPR Measurement Procedure

SPR angle scans covered an external incidence angle range of 48º to 66º for liquid measurements. The fluid cell was filled using a polypropylene syringe, and polypropylene stoppers were wedged into the injection ports to seal the cell and prevent evaporation (this arrangement is effective also for volatile solvents, e.g., tetrahydrofuran[24]). After mounting a sensor chip, angle scan measurements followed a defined sequence, whereby measurements for index-matched solvent

samples were undertaken prior to measurements for glycine solutions. As quality control, we repeated measurements of all solutions using the same as well as different SPR chips (N = 9-13 repeats across 10 Au chips, Tables S1 to S4, and N = 10-12 across 6 Au+PS chips, Table S2, S4 and S5). Before measurement of any individual liquid composition in the sequence, the liquid cell was flushed and rinsed with DI water three times. Then, the liquid cell was further flushed three times with the sample of interest and the fourth refill was held in the liquid cell for three minutes before the data scan was taken. Upon completing a measurement sequence, the Au chip and all parts of the liquid cell setup were removed from the prism and cleaned by the aforementioned 1% Hellmanex protocol. All angle shifts between solvents and glycine solutions were reversible.

S6.6. SPR Data Analysis

SPR angle scan spectra were analyzed using Winspall [25], a freely available software widely used over more than 20 years for studies of interfacial systems from self-assembled monolayers and Langmuir-Blodgett ultrathin films to protein biosensing [23, 24, 26, 27]. It computes the theoretical reflectivity (i.e., reflected intensity normalized by the incidence intensity) at each incidence angle due to light propagation through the SPR system, which is considered a stack of material layers in the optical analysis (i.e., glass prism/sensor chip, Cr adhesion layer, Au coating, PS coating, if present, concentrated interfacial liquid layer, if present, and bulk liquid medium above the surface) (**Figure S7**). In such systems, the reflectivity is completely determined by each layer's n_r and thickness (t), assuming perfectly flat interfaces, which is justified by the precision ground SPR chips and prism used, and by the sub-wavelength nature of the nanoscale layers involved. By comparing calculated plots of reflectivities against incidence angle (θ) with measured spectra, it can be determined if the n_r and t values used in Winspall matched the experimental results. If not, these layer parameters are adjusted to obtain good fit between the calculated and observed reflectivities.

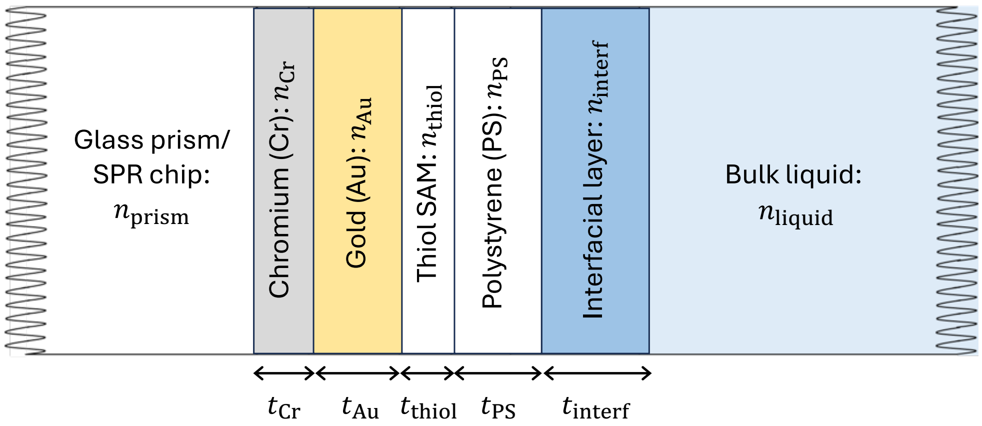

***Figure S7**. Schematic of the constituent layers used in the optical model analysis of the experimental SPR system. All measurements/models include glass prism, chromium (Cr), gold (Au) and bulk liquid layers. Measurements on polystyrene (PS) coated Au chips also include thiol and PS layers. Glycine solution measurements can also include an interfacial layer, if present. Each layer is described by its refractive index and thickness, except for the macroscopic glass and bulk liquid layers, which are described only by n_r values because of their sizes are macroscopic compared to the thin nanoscale interfacial layers.*

For the present experiments, measurements for each SPR chip were performed as successive layers were added and corresponding Winspall "models" were analyzed to determine each layer's n_r and t values. Angle scans were first measured with the liquid cell filled only with a pure solvent or index matched solution (i.e., water, methanol, 1-propanol or methanol:1-propanol mixtures). Since n_r of the LaSFN9 glass used for the prism/sensor chip is specified at high precision according to industry standard (n_r = 1.8455), n_r of the bulk liquid could be determined from Snell's Law that relates the n_r values to the reflectivity "edge" seen in angle scans (see Figure 2 in main text), i.e., the critical angle of total internal reflection ($\theta_{TIR} = \sin^{-1} n_{liquid}/n_{glass}$). The measured bulk liquid values are shown in **Table S1** and **Table S2** and corroborated by literature values (**Table S3** and **Figure S1**). Next, the Au and Cr parameters were obtained (**Table S4**): i) thickness was determined by matching the slopes in reflectivity plots around θ_{TIR} (thinner metal layers are more transparent and have lower reflectivity); and ii) n_r of Au could be identified from the angle and width of the reflectivity minimum indicating surface plasmon coupling (θ_{SPR})—plasmon propagation is modified by the optical density and damping of Au as described by its n_r, and the Cr adhesion layer is so thin that its exact n_r value has no effect on matching the SPR data (a standard value consistent with literature was used). Subsequently, if a PS coating is present, SPR scans were taken after each of the thiol deposition and PS spin coating steps to determine their thickness from further shifts in θ_{SPR} (literature values of n_r = 1.46 and 1.59 for thiol SAM and PS, respectively, were assumed.)[28, 29]

S7. Molecular Simulations Detail

We note that even the thickest films studied here exhibit a depletion of glycine in the central region of the film away from the solid and vacuum interfaces. We expect that for thick enough films the reservoir of molecules available will be large enough so that there will be no depletion in this central region, and that the interfacial excess will reach a limiting value with increasing film thickness. **Figure S8** shows how the interfacial concentration in a 1 nm region depends on the inverse film thickness. Based on this we can estimate that for very thick films where the depletion would be insignificant, the interfacial concentration is expected to be between 1.0 and 1.1 g cm^{-3}. This value corresponds 8-9 glycine molecules per 1 nm^3.

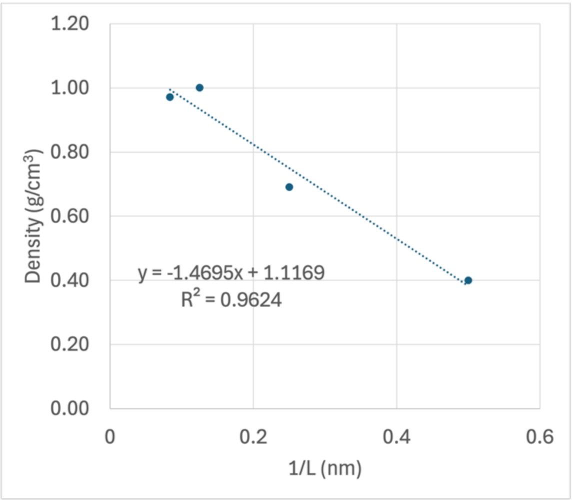

Figure S8. Dependence of interfacial concentration averaged over a 1 nm region vs inverse film thickness.

References

(1) Tilton, L. W. a. T., J.K. Refractive Index and Dispersion of Distilled Water for Visible Radiation, at temperatures 0-60°C. *Journal of Research of the National Bureau of Standards* **1938**, *20* (4), 419-477.

(2) Daimon, M.; Masumura, A. Measurement of the refractive index of distilled water from the near-infrared region to the ultraviolet region. *Appl. Optics* **2007**, *46* (18), 3811-3820. DOI: 10.1364/AO.46.003811.

(3) Chang, H.-J.; Munera, N.; Lopez-Zelaya, C.; Banerjee, D.; Beadie, G.; Van Stryland, E. W.; Hagan, D. J. Refractive index measurements of liquids from 0.5 to 2 um using Rayleigh interferometry. *Opt. Mater. Express* **2024**, *14* (5), 1253-1267. DOI: 10.1364/OME.519907.

(4) El-Kashef, H. The necessary requirements imposed on polar dielectric laser dye solvents. *Physica B: Condensed Matter* **2000**, *279* (4), 295-301. DOI: https://doi.org/10.1016/S0921-4526(99)00856-X.

(5) Kozma, I. Z.; Krok, P.; Riedle, E. Direct measurement of the group-velocity mismatch and derivation of the refractive-index dispersion for a variety of solvents in the ultraviolet. *J. Opt. Soc. Am. B* **2005**, *22* (7), 1479-1485. DOI: 10.1364/JOSAB.22.001479.

(6) Rheims, J.; Köser, J.; Wriedt, T. Refractive-index measurements in the near-IR using an Abbe refractometer. *Measurement Science and Technology* **1997**, *8* (6), 601. DOI: 10.1088/0957-0233/8/6/003.

(7) O'Brien, R. N.; Quon, D. Refractive index of some alcohols and saturated hydrocarbons at 6328 A. *Journal of Chemical & Engineering Data* **1968**, *13* (4), 517-517. DOI: 10.1021/je60039a017.

(8) Johnson, P. B.; Christy, R. W. Optical constants of transition metals: Ti, V, Cr, Mn, Fe, Co, Ni, and Pd. *Physical Review B* **1974**, *9* (12), 5056-5070. DOI: 10.1103/PhysRevB.9.5056.

(9) Corson, D. R.; Lorrain, P. *Introduction to electromagnetic fields and waves*; W.H. Freeman, 1962.

(10) Jones, F. E.; Harris, G. L. ITS-90 density of water formulation for volumetric standards calibration. *Journal of Research of the National Institute of Standards and Technology* **1992**, *97* (3), 340.

(11) Houck, R. C. A Note on the Density of Glycine. *Journal of the American Chemical Society* **1930**, *52* (6), 2420-2420. DOI: 10.1021/ja01369a501.

(12) Dawson, A.; Allan, D. R.; Belmonte, S. A.; Clark, S. J.; David, W. I. F.; McGregor, P. A.; Parsons, S.; Pulham, C. R.; Sawyer, L. Effect of High Pressure on the Crystal Structures of Polymorphs of Glycine. *Crystal Growth & Design* **2005**, *5* (4), 1415-1427. DOI: 10.1021/cg049716m.

(13) T. Sivanesan, V. N. a. S. P. Non-linear optical properties of α-glycine single crystals by Z-Scan technique. *Indian Journal of Science and Technology* **2010**, *2010*. DOI: https://dx.doi.org/10.17485/ijst/2010/v3i6.5.

(14) Guerin, S.; Stapleton, A.; Chovan, D.; Mouras, R.; Gleeson, M.; McKeown, C.; Noor, M. R.; Silien, C.; Rhen, F. M. F.; Kholkin, A. L.; et al. Control of piezoelectricity in amino acids by supramolecular packing. *Nat. Mater.* **2018**, *17* (2), 180-+, Article. DOI: 10.1038/nmat5045.

(15) Priya, N. S. Solution growth of γ-glycine from cadmium sulphate as solvent for frequency doubling applications. *Materials Research Express* **2019**, *6* (9), 095101. DOI: 10.1088/2053-1591/ab2dc9.

(16) Ashok Kumar, R.; Ezhil Vizhi, R.; Sivakumar, N.; Vijayan, N.; Rajan Babu, D. Crystal growth, optical and thermal studies of nonlinear optical γ-glycine single crystal grown from lithium nitrate. *Optik* **2012**, *123* (5), 409-413. DOI: https://doi.org/10.1016/j.ijleo.2011.04.019.

(17) Sivakumar, N.; Jayavel, R.; Anbalagan, G.; Yadav, R. R. Synthesis, growth, spectral, electrical, mechanical and thermal characterization of a potential optical material: γ-glycine single crystal. *Optical Materials* **2018**, *80*, 177-185. DOI: https://doi.org/10.1016/j.optmat.2018.04.051.
(18) Goodwin, R. D. Methanol Thermodynamic Properties From 176 to 673 K at Pressures to 700 Bar. *J. Phys. Chem. Ref. Data* **1987**, *16* (4), 799-892. DOI: 10.1063/1.555786 (acccessed 11/14/2025).
(19) Janz, G. J.; Tomkins, R. P. T. *Nonaqueus Electrolytes Handbook, Vol. 1*; Academic Press, New York, 1972.
(20) Hevia, F.; Alonso, V.; Cobos, A.; González, J. A.; Sanz, L. F.; García de la Fuente, I. Density, speed of sound, refractive index and relative permittivity of methanol, propan-1-ol or pentan-1-ol + benzylamine liquid mixtures. Application of the Kirkwood-Fröhlich model. *The Journal of Chemical Thermodynamics* **2022**, *168*, 106737. DOI: https://doi.org/10.1016/j.jct.2022.106737.
(21) Hales, J. L.; Ellender, J. H. Liquid densities from 293 to 490 K of nine aliphatic alcohols. *The Journal of Chemical Thermodynamics* **1976**, *8* (12), 1177-1184. DOI: https://doi.org/10.1016/0021-9614(76)90126-9.
(22) Soto, A.; Arce, A.; Khoshkbarchi, M. K. Effect of cation and anion of an electrolyte on apparent molar volume, isentropic compressibility and refractive index of glycine in aqueous solutions. *Biophysical Chemistry* **1999**, *76* (1), 73-82. DOI: 10.1016/s0301-4622(98)00223-3.
(23) Knoll, W. Interfaces and thin films as seen by bound electromagnetic waves. *Annual Review of Physical Chemistry* **1998**, *49*, 569-638. DOI: 10.1146/annurev.physchem.49.1.569.
(24) Lau, K. H. A.; Duran, H.; Knoll, W. In situ Characterization of N-Carboxy Anhydride Polymerization in Nanoporous Anodic Alumina. *J. Phys. Chem. B* **2009**, *113* (10), 3179-3189. DOI: 10.1021/jp809593d.
(25) *Winspall Data Analysis Software*. Resonant Technologies GmbH, http://www.res-tec.de/downloads.html (accessed 2021 February).
(26) Lau, K. H. A.; Huang, C.; Yakovlev, N.; Chen, Z. K.; O'Shea, S. J. Direct adsorption and monolayer self-assembly of acetyl-protected dithiols. *Langmuir* **2006**, *22* (7), 2968-2971. DOI: 10.1021/la053248g.
(27) Halay, E.; Bozkurt, S.; Capan, R.; Erdogan, M.; Unal, R.; Acikbas, Y. Calix 4 arene-triazine conjugate intermediate: optical properties and gas sensing responses against aromatic hydrocarbons in Langmuir-Blodgett films. *Research on Chemical Intermediates* **2020**, *46* (10), 4433-4445. DOI: 10.1007/s11164-020-04213-9.
(28) Lide, D. R., G.W.A. Milne. *Handbook of Data on Organic Compounds.*; CRC Press, Inc., 1994.
(29) Sultanova, N.; Kasarova, S.; Nikolov, I. Dispersion Properties of Optical Polymers. *Acta Physica Polonica A* **2009**, *116*, 585-587. DOI: 10.12693/APhysPolA.116.585.
