# Supplementary material for: Interface-Induced Concentration Enhancement in Glycine Solutions Investigated Using Surface Plasmon Resonance Spectroscopy and Molecular Dynamics Simulations
Source: J Phys Chem Lett. 2026 May 4;17(19):5535–40. doi: 10.1021/acs.jpclett.5c03688 (PMC13181769; doi:10.1021/acs.jpclett.5c03688)
Supplement: Supplementary file 2 [file jz5c03688_si_002.pdf]

jz-2025-036887.R1

Name: Peer Review Information for "Interface-Induced Concentration Enhancement in Glycine Solutions Investigated Using Surface Plasmon Resonance Spectroscopy and Molecular Dynamics Simulations"

First Round of Reviewer Comments

Reviewer: 1

Comments to the Author

In this work, the authors use surface plasmon resonance (SPR) spectroscopy and molecular dynamics (MD) simulations to demonstrate that glycine concentration is enhanced at gold and polystyrene interfaces in undersaturated aqueous solutions. The observation of enrichment at both surfaces suggests the effect is not specific to surface chemistry or topography, and the authors attribute the enhancement to non-specific van der Waals interactions. They claim this is the first direct experimental evidence of interfacial concentration enhancement in such systems.

The structure and composition of interfacial solution layers are central to phenomena such as catalysis, crystallization and self-assembly. This study therefore represents a meaningful contribution and should be of broad interest to the readership of JPCL. However, I suggest the authors consider the questions and comments below.

The authors are appropriately cautious in their novelty claim. On page three, they state: "However, to the best of our knowledge, an interfacial concentration effect has not yet been directly experimentally observed or quantified in small molecule liquid mixtures, such as aqueous glycine solutions." This framing is fair. However, there is prior direct experimental evidence of enhanced amino acid concentration at the air–water interface (e.g., https://pubs.acs.org/doi/10.1021/acs.jpclett.2c00231), as well as at interfaces involving miscible liquids and vapor (e.g., https://pubs.acs.org/doi/full/10.1021/jp011190i). There are also computational and experimental studies of concentration enhancement in ionic

and polar solutions at charge-neutral interfaces. While these systems differ from the one studied here, a comparison would help clarify the specific novelty of the current results.

Related to this, the attribution of glycine enrichment solely to van der Waals interactions feels speculative. What direct evidence supports this claim? Have the authors considered whether entropic driving forces might also play a role in the observed enrichment?

In connection with this point, the MD simulations suggest that concentration enhancement occurs over a region on the order of 1 nm near the surface. However, it is not clear how the interaction parameters between glycine and the model wall were chosen. Since the strength of the interaction will directly influence the degree of enrichment and the extent of structural deviation from bulk solution, additional discussion is warranted. For example, if the surface–solute interactions were weakened, how would the interfacial profile and excess density change? Exploring this parameter space could help support the authors' interpretation of the driving forces behind glycine enrichment.

On page one, the authors state that studies of crystal nucleation have not considered the intrinsic properties of materials in contact with the solution. This overlooks prior computational studies that have investigated model solid surfaces and their influence on nucleation mechanisms and rates. A more accurate framing might acknowledge this body of work while emphasizing the need for experimental validation.

Reviewer: 2

Comments to the Author

REVIEW on jz-2025-036887

In the submitted work, Mackay and co-workers present a novel study of glycine at solidliquid interface using a combination of MD simulation and SPR experimental measurements. The increased concentration of glycine nearer the interface is indeed intriguing. Overall, the reviewer is satisfied with the novelty of the work and that the conclusion is supported by the comprehensive range of results.

The manuscript would be recommended for publication subject to that the authors could address the following points:

- A generic LJ wall was used in the computational study – did the authors consider that there is no surface effect at all? If one pictures the system studied as a surface adsorption process (glycine dissolved in water), such process would certainly be influenced by the nature and magnitude of the binding energy between the active (glycine) and the solid substrate.
- Results of the MD study (figure 1b) suggests that there is barely any glycine molecules beyond 1 or 2 nm – does this imply that most of the glycine molecules would saturate at the interface?
- Did the authors consider the interaction between glycine and water in the present work, which was discussed in some publications, e.g. Di Gioacchino et al. J. Mol. Liquids 301, 2020, 112407
- For SPR experiments, any surface characterisation as quality control?
- It might be useful to carry out a kinetics study using SPR rather than the equilibrium state?

Author's Response to Peer Review Comments:

Please see attached point by point response to reviewer comments

We would like to thank both reviewer for taking the time to review the manuscript and to provide us insightful comments and feedback.

Reviewer: 1

Recommendation: This paper may be publishable, but major revision is needed; I would like to be invited to review any future revision.

Comments:
In this work, the authors use surface plasmon resonance (SPR) spectroscopy and molecular dynamics (MD) simulations to demonstrate that glycine concentration is enhanced at gold and polystyrene interfaces in undersaturated aqueous solutions. The observation of enrichment at both surfaces suggests the effect is not specific to surface chemistry or topography, and the authors attribute the enhancement to non-specific van der Waals interactions. They claim this is the first direct experimental evidence of interfacial concentration enhancement in such systems.

The structure and composition of interfacial solution layers are central to phenomena such as catalysis, crystallization and self-assembly. This study therefore represents a meaningful contribution and should be of broad interest to the readership of JPCL. However, I suggest the authors consider the questions and comments below.

We thank the reviewer for their positive comments about the manuscript and address the specific questions and comments below.

The authors are appropriately cautious in their novelty claim. On page three, they state: "However, to the best of our knowledge, an interfacial concentration effect has not yet been directly experimentally observed or quantified in small molecule liquid mixtures, such as aqueous glycine solutions." This framing is fair. However, there is prior direct experimental evidence of enhanced amino acid concentration at the air–water interface (e.g., https://pubs.acs.org/doi/10.1021/acs.jpclett.2c00231), as well as at interfaces involving miscible liquids and vapor (e.g., https://pubs.acs.org/doi/full/10.1021/jp011190i). There are also computational and experimental studies of concentration enhancement in ionic and polar solutions at charge-neutral interfaces. While these systems differ from the one studied here, a comparison would help clarify the specific novelty of the current results.

We thank the reviewer for this comment, and we have now included these prior experimental evidence examples in the introduction, highlighting that our study's focus on concentration enhancement in mixtures at solid surfaces. The section now reads:

> *"Experimental studies of binary metal alloy interfaces[13] and polymer solutions at solid surfaces[14] have been reported to show interfacial heterogeneity and surface enrichment. Enrichment of some small molecule mixtures at liquid-vapour interfaces has been observed from direct measurements, such as amino acids at water-air interfaces[15] and water-alcohol mixtures at air interfaces.[16, 17] However, to the best of our knowledge, an interfacial concentration enhancement effect for small molecules in liquid solutions at solid surfaces has not been directly observed experimentally or quantified previously."*

Related to this, the attribution of glycine enrichment solely to van der Waals interactions feels speculative. What direct evidence supports this claim? Have the authors considered whether entropic driving forces might also play a role in the observed enrichment?

Our earlier simulations of glycine aqueous solutions at Lennard Jones (LJ) walls (representing van der Waals interactions) (currently ref.6: McKechnie et al., 2022: https://doi.org/10.1021/acs.jpcc.2c04410) found that the concentration enhancement of glycine at the interface depended strongly on the LJ parameters and thus did not require electrostatic interactions. The following statement in the introduction:

> *"This was unexpected because these surfaces do not have a specific affinity with glycine, and the observations were attributed to omnipresent van der Waals interactions, which in turn lead to the formation of a nanoscale layer of highly concentrated glycine solution at hydrophobic interfaces.[2] "*

refers to what was reported in the earlier work by McKechnie et al. 2022 (originally ref.5, now ref.6, which includes coauthors Johnston and Sefcik of this manuscript). It does not mean to imply that electrostatic interactions do not play a role in general (which of course would be significant in charged or highly polar systems), but rather that they are not necessary for the concentration enhancement to occur. In fact, it was found that electrostatic interactions were not the dominant effect at the interface between a glycine solution and tridecane/PTFE (*ibid.*). Regarding entropic effects, the interaction with the surface clearly overcomes the entropic effects of mixing, leading to the concentration enhancement.

Therefore, we agree with the referee that electrostatic and entropic effects will play a role, and that the overall concentration enhancement will depend on the interplay of all interactions including van der Waals, electrostatic and entropic effects. To avoid misinterpretation, we have rephrased this statement as follows:

> *"This was unexpected because these surfaces do not have a specific affinity with glycine, and the observations were mainly attributed to van der Waals interactions, which in turn led to the formation of a nanoscale layer of highly concentrated glycine solution at hydrophobic interfaces.[2] "*

In connection with this point, the MD simulations suggest that concentration enhancement occurs over a region on the order of 1 nm near the surface. However, it is not clear how the interaction parameters between glycine and the model wall were chosen. Since the strength of the interaction will directly influence the degree of enrichment and the extent of structural deviation from bulk solution, additional discussion is warranted. For example, if the surface–solute interactions were weakened, how would the interfacial profile and excess density change? Exploring this parameter space could help support the authors' interpretation of the driving forces behind glycine enrichment.

As discussed in the previous point, our previous work (McKechnie et al., 2022) investigated the effect of surface interaction on the concentration enhancement in supersaturated glycine solutions, for a range of Lennard Jones (LJ) parameters. In the present manuscript, we show that the enhancement is also predicted in saturated glycine solutions, corresponded to the highest concentration experimentally investigated in this manuscript.

We selected LJ parameters in the middle of the range explored by McKechnie et al. and considered simulated liquid films over 10 nm, finding glycine interface widths of the order of 1 nm. The exact interfacial width will depend on the values of the LJ parameters, and we acknowledge that finite size effects are at play, with the glycine amassing at the surface and consequently becoming depleted in the centre of the film. Significantly thicker films would be required to enable the glycine to reach the limiting interfacial concentration enhancement corresponding to contact with bulk glycine solution and make a more accurate estimate of the interface width. Nevertheless, the simulations serve to illustrate the effect of a surface on glycine solution structure and interfacial concentration enhancement.

Moreover, we note that our experimental results do not depend on an accurate estimate of the interfacial thickness, as the measurements are sensitive to the overall change in glycine mass at the interface via changes in the local refractive index. The final experimental results (Figure 3c and d) gave the excess surface mass density (i.e., excess glycine mass per unit area), which is independent of the interfacial width estimated from MD simulations (explanation of the experimental surface mass derivation is described in detail in SI section S5 and Figures S4 and S5).

On page one, the authors state that studies of crystal nucleation have not considered the intrinsic properties of materials in contact with the solution. This overlooks prior computational studies that have investigated model solid surfaces and their influence on nucleation mechanisms and rates. A more accurate framing might acknowledge this body of work while emphasizing the need for experimental validation.

We think the reviewer is referring to the following paragraph on page 3 of the manuscript:

> *" Studies of crystal nucleation have extensively explored interfacial effects based either on surface structure or topography[2, 3] or on surface chemical properties.[2-4] However, these investigations have focused on specific surface functional groups rather than the intrinsic properties of a surface in contact with a solution.[4]"*
>
> (2) McKechnie, D.; Anker, S.; Zahid, S.; Mulheran, P. A.; Sefcik, J.; Johnston, K. Interfacial Concentration Effect Facilitates Heterogeneous Nucleation from Solution. *Journal of Physical Chemistry Letters* 2020, *11* (6), 2263-2271. DOI: 10.1021/acs.jpclett.0c00540.
>
> (3) Vesga, M. J.; McKechnie, D.; Mulheran, P. A.; Johnston, K.; Sefcik, J. Conundrum of γ glycine nucleation revisited: to stir or not to stir? *CrystEngComm* 2019, *21* (13), 2234-2243, 10.1039/C8CE01829D. DOI: 10.1039/C8CE01829D.
>
> (4) Boyes, M.; Alieva, A.; Tong, J.; Nagyte, V.; Melle-Franco, M.; Vetter, T.; Casiraghi, C. Exploiting the Surface Properties of Graphene for Polymorph Selectivity. *ACS Nano* 2020, *14* (8), 10394-10401. DOI: 10.1021/acsnano.0c04183.

This sentence was referring to the experimental work on nucleation from solution that typically has focused on surface functionalisation and topographical effects, including epitaxial matching and confinement. We acknowledge that the originally cited papers were not particularly representative, and we would like to change the citations as follows:

> *"Experimental studies of crystal nucleation have extensively explored interfacial effects based either on surface structure or topography[2, 3] or on surface functionalization,[4, 5] rather than the intrinsic properties of a material in contact with a solution.[4, 6]"*

(2) Epitaxy: a methodological approach to the study of an old phenomenon, M. Bruno, L. Pastero, A. Cotellucci and D. Aquilano, 10.1039/D2CE00340F (Highlight) CrystEngComm, 2022, **24**, 4165-4173

(3) Crystallization and Polymorphism under Nanoconfinement, Noalle Fellah, Isaac Jerome C. Dela Cruz, Bryan G. Alamani, Alex G. Shtukenberg, Ajinkya V. Pandit, Michael D. Ward, and Allan S. Myerson, *Crystal Growth & Design* 2024 *24* (8), 3527-3558; 10.1021/acs.cgd.3c01082

(4) Boyes, M.; Alieva, A.; Tong, J.; Nagyte, V.; Melle-Franco, M.; Vetter, T.; Casiraghi, C. Exploiting the Surface Properties of Graphene for Polymorph Selectivity. *ACS Nano* 2020, *14* (8), 10394-10401. DOI: 10.1021/acsnano.0c04183.

(5) Role of Self-Assembled Surface Functionalization on Nucleation Kinetics and Oriented Crystallization of a Small-Molecule Drug: Batch and Thin-Film Growth of Aspirin as a Case Study, Fiora Artusio, Francesco Fumagalli, Andrea Valsesia, Giacomo Ceccone, and Roberto Pisano, *ACS Applied Materials & Interfaces* 2021 *13* (13), 15847-15856; 10.1021/acsami.1c00460

(6) McKechnie, D.; Anker, S.; Zahid, S.; Mulheran, P. A.; Sefcik, J.; Johnston, K. Interfacial Concentration Effect Facilitates Heterogeneous Nucleation from Solution. *Journal of Physical Chemistry Letters* 2020, *11* (6), 2263-2271; 10.1021/acs.jpclett.0c00540.

Reviewer: 2

In the submitted work, Mackay and co-workers present a novel study of glycine at solid liquid interface using a combination of MD simulation and SPR experimental measurements. The increased concentration of glycine nearer the interface is indeed intriguing. Overall, the reviewer is satisfied with the novelty of the work and that the conclusion is supported by the comprehensive range of results. The manuscript would be recommended for publication subject to that the authors could address the following points:

We are pleased that the reviewer is satisfied with the novelty of the work and we have addressed the specific points below.

• A generic LJ wall was used in the computational study – did the authors consider that there is no surface effect at all? If one pictures the system studied as a surface adsorption process (glycine dissolved in water), such process would certainly be influenced by the nature and magnitude of the binding energy between the active (glycine) and the solid substrate.

While there may be specific binding interactions between a solid substrate and solute molecules, we show that these are not necessary to cause interfacial concentration enhancement and that non-specific LJ interactions with the surface are sufficient to induce this effect. Of course, this effect could be further enhanced by any specific interaction with surface functional groups or topological features.

Regarding a vacuum interface (approximating an air interface), there is a clear depletion of glycine at the vacuum interface, demonstrating that the interfacial concentration enhancement (or depletion) is interface-dependent. This is shown in the present simulations and also in our previous simulations for supersaturated solutions (currently ref.7: McKechnie et al. 2020 https://pubs.acs.org/doi/full/10.1021/acs.jpclett.0c00540).

As a control system, we also simulated bulk glycine solutions in which there were no surfaces present due to periodic boundary conditions. Our simulations used the previously tested force field by Cheong

and Boon, 2010 (currently ref.30: https://pubs.acs.org/doi/full/10.1021/cg100906s), which gave good properties for both the solution and alpha-glycine crystal polymorph.

• Results of the MD study (figure 1b) suggests that there is barely any glycine molecules beyond 1 or 2 nm – does this imply that most of the glycine molecules would saturate at the interface?

Yes, the reviewer is correct that in these simulations the glycine has amassed at the LJ wall interface and has become depleted in the rest of the liquid film. The stronger the interaction with the surface, the more glycine builds up, potentially reaching a density comparable to a glycine melt or crystal. This buildup of glycine at the interface causes a depletion of the molecules beyond 1-2 nm due to finite size effects. Given much larger simulation cells, there would be a larger reservoir of glycine molecules, and the depletion would be much less pronounced. It is however possible that with a larger reservoir of glycine, that there would be a larger concentration enhancement, and that the interfacial region would extend beyond the 1-2 nm range from the surface.

While this is a limitation of the simulations, we emphasise that this does not impact the experimental results, as the excess surface mass density reported in the end (i.e., excess glycine mass per area) is independent of the interface width, as discussed in a previous point replying to reviewer 1. Calculation of the excess surface mass density and a discussion about the independence from the interfacial width is presented in the Supplemental Information.

• Did the authors consider the interaction between glycine and water in the present work, which was discussed in some publications, e.g. Di Gioacchino et al. J. Mol. Liquids 301, 2020, 112407

We are aware of the long-standing discussion around formation of glycine clusters, at both the molecular level, and at the mesoscale level with one of the coauthors of this manuscript actively contributing to this topic:

- Jawor-Baczynska et al., 2013: https://doi.org/10.1039/C3FD00066D
- Zimbitas et al., 2019: https://doi.org/10.1016/j.colsurfa.2019.123633
- Sweatman et al., 2022: https://pubs.acs.org/doi/full/10.1021/acs.jpcb.2c01975

However, the nature and size of clusters is still very much an open question and is outside the scope of the present study.

As stated in a previous point, we used the force field previously validated by Cheong and Boon for glycine solutions and the alpha-glycine polymorph, although we do recognise that there is no guarantee this force field represents all aspects of the complex glycine-water interaction. Nevertheless, we think that the force field is sufficiently reliable to demonstrate that there is a buildup of glycine at the solid surface, and the simulation results should be qualitatively correct. We do however agree it is difficult to accurately quantify the buildup, not only because of glycine-water force field uncertainties and non-specificity of the LJ surface, but also due to the finite size effects mentioned earlier.

• For SPR experiments, any surface characterisation as quality control?

Surface quality control was exercised by two approaches—optical microscopy inspection to check for noticeable defects and exhaustive repetition of measurements on the same and across different SPR chips to verify molecular level (interfacial) measurement results. Some details are already alluded to in the main text, the Experimental and the SI section 6. However, the Experimental and the SI are now revised to consolidate and detail these strategies:

> Experimental: "*Details of materials and sample preparation methods are described in SI Section 6… Samples were checked for surface defects by brightfield and darkfield microscopy. Multiple repeated measurements were performed for each solution condition across different SPR chips (N = 9-13 across at least 10 Au chips and 6 PS coated chips) and the averages and standard errors are reported (see Tables S1 to S7).*"
>
> SI section 6.2 (sample preparation): "*Visual inspection by optical microscopy (Nikon Eclipse LV100) was performed on samplings of SPR chip batches (in brightfield to check for gross defects and in darkfield to reveal scattering from nanoscopic defects). Samples with noticeable surface defects were rejected… [PS] samples were dried for 24 hours in air and all chips were examined under an optical microscope (Leica M165 CM) to screen out chips with de-wetting defects before use. Samples with noticeable surface defects were rejected.*"
>
> SI section 6.5 (SPR measurement): "*As quality control, we repeated measurements of all solutions using the same as well as different SPR chips (N = 9-13 repeats across 10 Au chips, Tables S1 to S4, and N = 10-12 across 6 Au+PS chips, Table S2, S4 and S5). Before measurement of any individual liquid composition in the sequence, the liquid cell was flushed and rinsed with DI water three times. Then, the liquid cell was further flushed three times with the sample of interest and the fourth refill was held in the liquid cell for three minutes before the data scan was taken. Upon completing a measurement sequence, the Au chip and all parts of the liquid cell setup were removed from the prism and cleaned by the aforementioned 1% Hellmanex protocol. All angle shifts between solvents and glycine solutions were reversible*."

Brightfield microscopy can check for gross defects, and the darkfield can reveal scattering from nanoscopic defects. Samples with noticeable surface defects were rejected. Since molecular level issues are difficult to characterize in general (regardless of the type of microscopy, optical, scanning electron or atomic force), we relied on exhaustive rinsing and multiple repeated SPR measurements of glycine solutions and solvents on the same and different SPR sample chips to verify that the interfacial effects are genuine. All angle shifts between solvents and glycine solutions were reversible and the variances in the angle shifts for the same medium was small.

• It might be useful to carry out a kinetics study using SPR rather than the equilibrium State

The kinetics of formation of the interfacial concentration enhancement layer is anticipated to be very fast, as seen in the current MD simulations (nanosecond timescale) and when considering the high diffusion rate of small molecules such as glycine across very small distances involved in the current context. Conventional SPR kinetics measurements based on monitoring the change in reflected intensity have much lower time resolution (measuring changes in the seconds to minutes timescales) and they also cannot take into account the very large change in background medium refractive index (e.g., from water to >50 g/kg glycine in water) that will on its own also shift both the total internal reflection and

the plasmon coupling angle (hence we presented the parallel index-matched angle scan data for comparison). SPR angle tracking measurements have even slower time resolution. Developing a novel form of SPR or another novel evanescent surface technique applicable to dense solutions to measure such fast kinetics would be very challenging and is out of scope of the present study.

jz-2025-036887.R2

Name: Peer Review Information for "Interface-Induced Concentration Enhancement in Glycine Solutions Investigated Using Surface Plasmon Resonance Spectroscopy and Molecular Dynamics Simulations"

Second Round of Reviewer Comments

Reviewer: 1

Comments to the Author

I thank the authors for their responses to my queries and for the amendments made to the manuscript.

Thank you for the clarification regarding the attribution of enrichment to van der Waals forces. My earlier comment was not referring to the entropy of mixing of glycine in the bulk, but rather to solvent-mediated entropic effects at interfaces. In many systems, enrichment near nominally hydrophobic surfaces can arise from the release of interfacially constrained solvent molecules, leading to a net entropy gain even in the absence of a strong enthalpic attraction. Could the authors comment on whether such solvent-structuring effects were analysed or could be ruled out in their simulations?

Related to the above point, it would be helpful if the Lennard–Jones interaction parameters governing solute–surface and solvent–surface interactions were explicitly reported. I would also encourage the authors to comment in the main text on how the chosen parameters support the interpretation of the surface as having no particular affinity for glycine. As presented, the observed depletion of glycine from the bulk in favour of the interface appears consistent with a significant solute–surface attraction.

Author's Response to Peer Review Comments:

We thank the reviewer for these questions and have responded below and indicated changes in the manuscript to help to clarify these points.

Thank you for the clarification regarding the attribution of enrichment to van der Waals forces. My earlier comment was not referring to the entropy of mixing of glycine in the bulk, but rather to solvent-mediated entropic effects at interfaces. In many systems, enrichment near nominally hydrophobic surfaces can arise from the release of interfacially constrained solvent molecules, leading to a net entropy gain even in the absence of a strong enthalpic attraction. Could the authors comment on whether such solvent-structuring effects were analysed or could be ruled out in their simulations?

The LJ forces between solute and solvent atoms in the solution give rise to solute-solvent, solvent-solvent, and solute-solute interactions. In an interfacial system with a wall interface, the LJ forces give rise to additional wall-solute and wall-solvent interactions. In addition to these interactions, there are also configurational/demixing and orientational entropic effects. We fully agree with the referee that the resulting enrichment/depletion at the interface is a result of the complex interplay of these competing interactions. If we understand correctly, the reviewer is asking whether the increased concentration of glycine in the aqueous solution at the interface is due to the enthalpic wall interaction with the glycine, or whether it is due to an increase in entropy by releasing interfacially constrained water molecules. To try to answer this we consider our published and present results for glycine aqueous solutions at a vacuum (air) interface as well as at walls with various interaction strengths.

First, we consider a glycine-water solution at a vacuum (air) interface. In this case, we see a depletion of glycine at the interface, and conversely an enrichment of water molecules. This demixing effect indicates a lowering of configurational entropy. In addition to this, we see that glycine exhibits a weak orientational order at the interface (see Figure 6 of ref.6 in main text: McKechnie et al., J. Phys. Chem. C (2022) 10.1021/acs.jpcc.2c04410) which could mean that it is entropically favorable for glycine to remain in bulk solution where its orientational order is unconstrained. However, we have not measured the orientational order of water at the interface, so we cannot know whether the gain in orientational entropy of glycine is offset by a loss in orientational entropy of the water molecules. In addition, the total glycine-glycine and glycine-water potential energy in the bulk mixture would be reduced if the glycine was at the interface. Overall, the depletion in this case could be caused by several factors and we are unable to say which interaction is the major factor in causing the depletion of glycine at the vacuum interface. However, this is not a topic of the present manuscript.

Next, we consider Figure 8 in the McKechnie et al., 2022, paper (*ibid*.), which shows the density profiles of water and glycine solutions at wall potentials with a range of sigma and epsilon values for the LJ wall. For the weakest wall interactions, we see a similar effect as for the vacuum interface, although the orientational order of glycine is stronger at the wall, compared to the vacuum interface. As for the case of the vacuum interface, we cannot say whether the depletion of glycine at the interface is caused by an increase in orientational entropy of glycine or by avoidance of a loss of potential energy at the interface.

Finally, we consider what happens when we increase the wall LJ interaction strength (again refer to Figure 8 lower graphs where the value of epsilon is higher). We note that increasing this parameter affects both the water-wall and glycine-wall interactions. Now we can see that

as epsilon is increased the concentration of glycine at the interface also increases. From the above arguments, it can be deduced that

(a) the interaction of the wall must overcome the orientational entropy of glycine as glycine becomes highly ordered at the surface
(b) the effects of releasing water from the wall interface would likely be offset by constraining the glycine at the wall into a glycine rich layer, and
(c) there is no loss of bulk glycine-glycine or glycine-water potential energy by having glycine at the wall interface as this is compensated by the glycine-wall interaction.

For (b) we recognise that it is possible that the entropy at the interface may be higher for intermediate wall strengths where both glycine and water are present at the interface. However, since the interfacial glycine concentration increases with the wall strength (up to saturation) the enthalpic glycine-wall potential contribution is clearly the driving force for the interfacial concentration enhancement of glycine in aqueous solutions. We believe that it is glycine's stronger van der Waal's interactions, compared to water, that lead to the enhancement at an interface with strong van der Waal's interactions, i.e., the glycine-wall interaction outcompetes the other effects.

We note that this effect could be different in other solutions, as it will depend on the relative van der Waals interactions of the solute and solvent molecules with each other, as well as with the surface, in conjunction with interfacial entropic effects. For this reason, we cannot a priori say whether a given solute-solvent-surface will exhibit an enhancement or depletion at the surface. For example, several studies have shown that ethanol-water mixtures have an enrichment of ethanol molecules at vacuum (air) interfaces [see, for example, Hyde et al., J. Mol. Liq. (2019). https://doi.org/10.1016/j.molliq.2019.111005. Now ref.26 in the manuscript].

We recognise that this is a complex issue and deserves further discussion in the manuscript. To address this point, on page 8 we have:

- removed the sentence: "The van der Waals interactions of the surface with the molecules are responsible for generating the peaks in the density profiles near the solid-liquid interface."
- added the paragraph: “We note that the interfacial concentration effect observed here could be different for other solution-surface combinations, as it will depend on the relative van der Waals interactions of the solute and solvent molecules with each other, as well as with the surface, in conjunction with interfacial entropic effects. For example, for glycine aqueous solution at a vacuum (air) interface there is a depletion of glycine at the interface[6] whereas for ethanol-water mixtures there is an enrichment of ethanol molecules at the vacuum (air) interface.[25] For this reason, we cannot a priori say whether a given solute-solvent-surface will exhibit an enhancement or depletion at the surface. Nevertheless, our current understanding is that in glycine aqueous solutions, it is glycine's stronger van der Waal's interaction, compared to water, that is the primary driving force for the interfacial enhancement of glycine at the surfaces studied.”

Related to the above point, it would be helpful if the Lennard–Jones interaction parameters governing solute–surface and solvent–surface interactions were explicitly reported. I would also encourage the authors to comment in the main text on how the chosen parameters support the interpretation of the surface as having no particular

affinity for glycine. As presented, the observed depletion of glycine from the bulk in favour of the interface appears consistent with a significant solute–surface attraction.

In the submitted manuscript, we reported the LJ parameters for the wall interaction in the Computational Methods section on page 18 in the first paragraph:

> "Glycine was represented using the generalized AMBER Force Field (GAFF) with CNDO charges, and water was represented using the SPC/E water model as in previous studies.[7, 30] The model surface was represented by a Lennard Jones (LJ) wall potential with σ_{LJ} = 0.34 nm and ε_{LJ} = 10.0 kcal mol^{-1}.[6]"

The wall parameters are within the range of parameters used to represent heptane, tridecane and graphite (ref.6 of manuscript). The actual van der Waals interaction parameters for the solvent and solute with the wall are then determined using standard mixing rules, as implemented in LAMMPS.

In addition, the data set cited in Associated Content includes the input files used for the LAMMPS simulations, which will enable readers to reproduce the simulations presented. Unfortunately, the dataset DOI is assigned but will not be active until after our manuscript is published when no further changes to the dataset are required. If the reviewer wishes to review this data now, we can provide a different means of doing so.

We have modified the methodology to reflect this information:

> "Glycine was represented using the generalized AMBER Force Field (GAFF) with CNDO charges, and water was represented using the SPC/E water model as in previous studies.[7, 30] The model surface was represented by a Lennard Jones (LJ) wall potential with σ_{LJ} = 0.34 nm and ε_{LJ} = 10.0 kcal mol^{-1}, which are within the range of LJ parameters determined for heptane, tridecane, and graphite surfaces.[6]… The LAMMPS input files are provided open access (see Associated Content)."

jz-2025-036887.R3

Name: Peer Review Information for "Interface-Induced Concentration Enhancement in Glycine Solutions Investigated Using Surface Plasmon Resonance Spectroscopy and Molecular Dynamics Simulations"

Third Round of Reviewer Comments

Reviewer: 1

Comments to the Author

I thank the authors for carefully considering the points raised in my previous review.

The revised discussion of the driving forces for solute accumulation at the interface is now much more balanced.

With regard to the reply concerning the chosen intermolecular parameters, an important methodological detail is currently absent from the manuscript and should be included. Specifically, the statement:

“The actual van der Waals interaction parameters for the solvent and solute with the wall are then determined using standard mixing rules, as implemented in LAMMPS.”

is essential for clarity and reproducibility.

While the provision of input files is appreciated, key methodological details such as this should be explicitly stated in the manuscript itself.

Author's Response to Peer Review Comments:

In response to the reviewer's request to provide information about the mixing rules, we have included additional information in the methodology section and the Supplementary Information explaining that we used modified mixing rules for the Lennard Jones interaction between the molecules and the wall, as discussed in our previous work (McKechnie, 2022). Specifically, we added the following sentence to the manuscript:

"The van der Waals interaction parameters for the solvent and solute with the LJ 9-3 wall were determined using modified Lorenz-Berthelot mixing rules, as described in our previous work.6"

Additionally, in reviewing these LJ epsilon parameters, we noticed some errors in the original simulation input files for the molecule-wall interactions. We have rerun the simulations with corrected parameters and note that this results in only small changes to the simulation results (e.g., total solution film width changed from 8.7 nm to 8.8 nm for simulation with 60 glycine and 1000 water molcules, and averaged interfacial concentration over the first 1 nm on the surface now reported as 1.0 g cm-3 vs. 1.4 g cm-3), and these do not alter the original qualitative analysis or conclusions of the manuscript. To reflect the changes, we have updated Figure 1 and Table 1 with the rerun data as well as made corresponding changes in the main text. For transparency, we have included a copy of the manuscript with all changes phighlighted.
